# Engineering modular intracellular protein sensor-actuator devices

**DOI:** 10.1038/s41467-018-03984-5

**Published:** 2018-05-14

**Authors:** Velia Siciliano, Breanna DiAndreth, Blandine Monel, Jacob Beal, Jin Huh, Kiera L Clayton, Liliana Wroblewska, AnneMarie McKeon, Bruce D. Walker, Ron Weiss

**Affiliations:** 1 Department of Synthetic and Systems Biology for Biomedicine, Istituto Italiano di Tecnologia, Via Morego 30, Genoa, 16163 Italy; 2 Department of Biological Engineering, Synthetic Biology Center, Massachusetts Institute of Technology, 500 Technology Square, Cambridge, MA 02139 USA; 3 Ragon Institute of Massachusetts General Hospital, Massachusetts Institute of Technology, and Harvard University, Cambridge, MA 02139 USA; 4 Howard Hughes Medical Institute, Chevy Chase, MA 20815 USA; 5 Raytheon BBN Technologies, Cambridge, MA 02138 USA; 6 Pfizer, Cambridge, MA 02139 USA; 7 Institute for Medical Engineering and Science, Massachusetts Institute of Technology, Cambridge, MA 02139 USA

## Abstract

Understanding and reshaping cellular behaviors with synthetic gene networks requires the ability to sense and respond to changes in the intracellular environment. Intracellular proteins are involved in almost all cellular processes, and thus can provide important information about changes in cellular conditions such as infections, mutations, or disease states. Here we report the design of a modular platform for intrabody-based protein sensing-actuation devices with transcriptional output triggered by detection of intracellular proteins in mammalian cells. We demonstrate reporter activation response (fluorescence, apoptotic gene) to proteins involved in hepatitis C virus (HCV) infection, human immunodeficiency virus (HIV) infection, and Huntington’s disease, and show sensor-based interference with HIV-1 downregulation of HLA-I in infected T cells. Our method provides a means to link varying cellular conditions with robust control of cellular behavior for scientific and therapeutic applications.

## Abstract (web summary)

Synthetic biology principles are often used to design circuits that tune gene expression in response to changes in intracellular environments. Here the authors design a modular platform for intracellular protein sensing devices with transcriptional output.

## Introduction

Synthetic biology can improve our understanding of rules underlying biological pathways^1,2^, and offer unique approaches to tackle a number of biomedical challenges such as cancer therapy^3^, metabolic diseases^4^, and antibiotic resistance^5^. Genetic circuits with therapeutic capabilities require tightly controlled output activation in response to dynamic changes in the intracellular environment. Endogenous inputs such as microRNAs^6,7^ and proteins^8–10^, whose level or state often correlate with the onset and progression of a disease, have been connected to circuitry function via transcriptional or translational regulation of chosen genes in mammalian cells. However, while circuits that tune output gene expression in response to specific miRNA signatures have been demonstrated^6^, a customizable framework to link intracellular protein sensing to programmed cellular responses still lags behind.

To this end, we report on our development of modular sensing-actuation devices that initiate programmed transcriptional response when detecting target intracellular proteins in mammalian cells. We demonstrate the modularity of this platform by creating devices that sense four different proteins associated with diseases and respond with either fluorescent reporter activation or biological activity where applicable (cell death or receptor downregulation). We envision these devices will find use in enabling protein-responsive therapeutic gene circuits as well as in basic research.

## Results

### A modular platform for intracellular protein detection

Our genetically encoded framework combines sensing and actuation modules (Supplementary Fig. 1). The sensing modules are based on intracellular antibodies (intrabodies) that have recently emerged as a new tool for therapeutic and functional genomics applications due to their ability to bind a wide range of proteins in several subcellular locations^11^. The actuation module takes advantage of the Tango-TEV technology, previously demonstrated to efficiently convert ligand/protein-induced dimerization into transcriptional output^12–14^(Supplementary Note 1, Supplementary Fig. 2). Thus, by coupling this sensing module with the Tango-TEV-derived actuation module, we extend this platform to detect intracellular, medically relevant proteins.

**Fig. 1:**
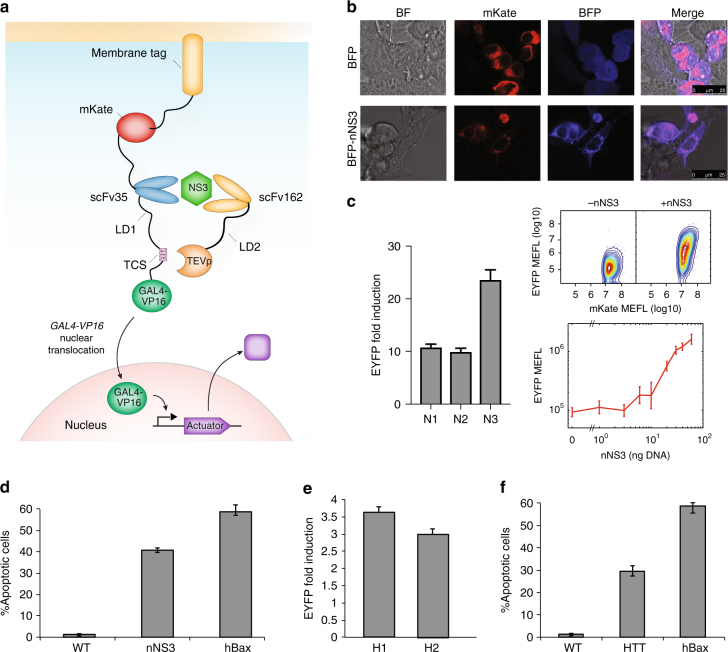
Protein sensing-actuation devices in mammalian cells. **a** Schematics of the protein sensor. One intrabody is anchored to the membrane and fused at the N-terminus to mKate fluorescent tag and at the C-terminus to the TEV cleavage site (TCS) and to a GAL4-VP16 transcriptional activator. A second intrabody is fused to the TEV protease (TEVp). Interaction of the intrabodies with the target protein results in TEVp-mediated release of membrane-anchored GAL4-VP16 and output activation. **b** Co-localization of BFP-nNS3 and mKate-scFv35-antibody in HEK293FT cells. Confocal images (×63) indicate co-localization when BFP is fused to nNS3 in HEK293FT cells (scale bar = 25 µm). Non-fused BFP was used as control and show diffused cellular localization. **c** Best performing variants of intrabody-TCS/intrabody-TEVp combinations (N1: scFv35-LD15-TCS(L)/TEVp-LD15-scFv162, N2: scFv35-LD0-TCS(L)/DD-scFv162-LD15-TEVp, N3: scFv35-LD0-TCS(L)/TEVp-LD0-scFv162) for nNS3 device. EYFP data shows fold induction and standard deviation using molecules of equivalent fluorescein (MEFL) of EYFP for cells expressing >1 × 10^7^ MEFL of transfection marker mKate. *n* = 3 independent technical replicates. Right-top inset: Two-dimensional flow cytometry plots for the best variant (N3) in the absence and presence of nNS3. Right-bottom inset: EYFP MEFL as function of the concentration of transfected DNA encoding nNS3 for the N3 variant of the device. **d** Selective cell death induced by pro-apoptotic hBax-N3 variant in cells expressing nNS3 was determined by Pacific-Blue conjugated to Annexin V staining 48 h posttransfection. Constitutively expressed CMV-hBax (labeled as “hBax”) was used as positive control for apoptosis. Data and standard error are of *n* = 3 independent technical replicates. **e** Best performing HTT (HDx-1 first exon with poliQ tract) devices (H1: Happ1-LD0-TCS(S)/DD-TEVp-LD0-Vl12.1, H2: Happ1-LD0-TCS(S)/TEVp-LD0-Vl12.1). Data shows EYFP fold induction and standard deviation of EYFP MEFL for cells expressing >3 × 10^7^ MEFL of transfection marker mKate. *n* = 3 independent technical replicates. **f** Selective cell death induced by hBax apoptotic protein in cells expressing HTT was determined by Pacific-Blue conjugated to Annexin V staining 48 h posttransfection. The assay was performed on HTT best performing device (Happ1-LD0-TCS(S)/DD-TEVp-LD0-Vl12.1) using the pro-apoptotic hBax as reporter activated by GAL4-VP16. Constitutively expressed CMV-hBax (labelled as “hBax”) was used as positive control for apoptosis. Data and s.e.m. are of *n* = 3 independent technical replicates

Specifically, one intrabody is fused at the N-terminus to a membrane-tethered fluorescent tag (mKate) and at the C-terminus to a Tobacco Etch Virus (TEV) cleavage site (TCS) and to a GAL4-VP16 transcriptional activator, forming a chimeric protein sequestered in the cytosol. A second intrabody is fused to the TEV protease (TEVp) that recognizes and cleaves the TCS (Supplementary Fig. 1). The presence of the target protein and subsequent binding of the two intrabodies results in TEVp cleavage of TCS and release of GAL4-VP16, which translocates into the nucleus and converts protein detection into programmed gene expression (Fig. 1a).

In order to optimize sensing-actuation performance, we designed variants of the device by tuning several of its features. TEVp was fused to the N-terminus or C-terminus of the intrabody with a Glycine-Serine flexible linker sequence. Moreover, to obtain devices with significant ON/OFF ratio for output expression, we tested flexible linker sequences of different length to maximize the likelihood of intrabodies with protein and TEVp with TCS interactions, and tested TCS mutants-TEVp complexes with different binding constants^15^ (Supplementary Note 2, Supplementary Fig. 3a, Supplementary Table 2).

We first tested the membrane tethered module of the device (membrane tag-mKate-sdAb19-GAL4-VP16, Supplementary Fig. 3a). Constitutive TEVp expression induced significant activation of the reporter gene (up to 100 fold ON/OFF induction), indicating that TEV cleavage site is accessible in the design configuration, and suggesting that careful tuning of protease expression is critical to maximize signal-to-noise ratio (Supplementary Fig. 3b, c).

The selection of target proteins for testing our sensing-actuation framework was based on: (i) partial or complete cytosolic localization, and (ii) existence of intrabodies binding two different epitopes of the antigen. Following these criteria, we engineered genetic devices that recognize NS3, HTT, and Tat/Nef proteins, respectively, specific for HCV, Huntington’s disease, and HIV.

### HCV sensor-actuator device

The HCV sensor-actuator device was designed to recognize NS3 serine protease^16^ (Fig. 1a). We engineered variants of the device architecture using single chain fragment intrabodies scFv35 and scFv162, previously reported to bind with high affinity to the N-terminus of NS3^17^ (nNS3) and to interfere with its viral activity. We confirmed NS3-intrabody interaction by fusing a BFP tag to the N-terminus of nNS3 (BPF-nNS3) (Fig. 1b). We then co-transfected membrane tethered LD15-TCS-S/L variants of the NS3 responsive devices along with TEVp-scFv162 driven by a constitutive promoter (hEF1α) in 293FT HEK cells which revealed nNS3-independent output activation (Supplementary Fig. 4a), thus confirming that protein-dependent signal induction requires appropriately-regulated TEVp activity (Supplementary Note 2). We fine-tuned TEVp expression transcriptionally and posttranslationally using a Tetracycline/Doxycycline responsive promoter (pTET) and a degradation domain tag (DD degron) regulated by the small molecule Shield. Basal pTET expression (in the absence of Doxycycline) enabled the device to be specifically activated by endogenous nNS3 concentrations in LD15-TCS-L (Supplementary Fig. 4b), but not LD15-TCS-S variant of the device (Supplementary Fig. 4c). Of note, while the best response is in the presence of rtTA3 but absence of Doxycycline and Shield, we show that nNS3 detection works even in absence of the transcriptional activator rtTA3 (−rtTA/−Dox/+Shield) (Supplementary Fig. 4b). This is probably due to the high sensitivity to the TEVp, since either leakiness of pTET combined with increased protein stability induced by Shield, or residual activity of rtTA3 in absence of Doxycycline^18,19^, are sufficient to induce output activation. Our findings that low affinity TEV cleavage site (TCS-L) and low sensor concentration provide the best operating conditions (Supplementary Note 3) are in agreement with the conclusions from a computational model we implemented of the system (Supplementary Note 5, Supplementary Fig. 15–19 and Supplementary Table 3).

We then engineered further nNS3 devices where the parameters described above were tuned, including a membrane-linked variant lacking a linker domain (LD0-TCS-L), and variants of TEVp-intrabody chimeric proteins in which the protease was fused to C- or N-terminus of scFv162 with or without a linker domain. These devices exhibited up to 24 EYFP fold induction (Fig. 1c, Supplementary Fig. 4d, e). Indeed, sensitive discrimination of target proteins requires concerted regulation of TEVp activity that relies on fine transcriptional regulation and TCS affinity. Increasing Dox levels resulted in an inability to discriminate NS3 expressing cells even when TCS-L was used, whereas fusing a DD domain to TEVp did not improve sensor performance either in TCS-L or TCS-S-based devices (Supplementary Fig. 4b,c).

We showed that reporter expression is a function of nNS3-encoding plasmid concentration by co-expressing various concentrations of nNS3 or BFP-nNS3 along with the device (Fig. 1c inset, Supplementary Fig. 5a). Interestingly, we observed that very small amounts of the BFP-nNS3 encoding plasmid (3–6 ng) resulted in strong activation of EYFP (Supplementary Fig. 5a–e), at intermediate transfection marker levels (10^6^ < mKate MEFL < 10^7^; 60 ng of DNA), EYFP induction was significantly higher in BFP-nNS3 than nNS3 expressing cells, while at high transfection marker levels (10^7^ < mKate MEFL < 10^8^) EYFP levels were comparable (Supplementary Fig. 5f). This is perhaps due to a higher stability of the protein conferred by BFP. When pro-apoptotic gene *hBax* was incorporated as output, specific induction of apoptosis was achieved in nNS3 expressing cells, demonstrating the device’s ability to rewire cellular fate (Fig. 1d, Supplementary Fig. 6).

The key parameters of circuit design inferred from the NS3 device (i.e., fine tuning of TEVp expression and affinity to cleavage site) were consequently considered in the development of additional sensing-actuator devices. We thus generated plasmid DNA backbones enabling facile interchange of protein-specific intrabodies, which allowed us to create and test new devices on a 2-week timeline.

### Huntington’s disease and HIV sensor-actuator devices

To verify that the platform can be customized to other relevant proteins, we next engineered devices for detecting Huntington’s disease and HIV infection. Huntington’s disease (HD) is a progressive, neurodegenerative disorder that results from expansion of a polyglutamine (polyQ) tract in the first exon (HDx-1) of huntingtin gene (HTT)^20^. We used previously developed scFvs (Happ1, V_L_12.3)^21^ and output response was either fluorescence or expression of *hBax*. We observed activation of fluorescence output and selective cell killing when HTT was present (Fig. 1e,f Supplementary Fig. 7a-c, Supplementary Fig. 8).

We then created devices to detect and respond to HIV infection by sensing Tat^22^ and Nef^23^ proteins. The HIV-1 transactivator protein Tat is a multifunctional protein that alters expression of host and viral genes, and plays a crucial role in HIV replication. The single chain fragments (scFv2, scFv3) used here, were previously designed to interfere with Tat activity^24^. The Tat-sensing devices engineered with these scFvs were able to induce reporter activation efficiently (Supplementary Fig. 9a-c). However, we did not perform a cell killing assay since Tat already exhibits mild apoptotic activity via the caspase-8 and Egr1-PTEN-FOXO3a pathways^25,26^.

**Fig. 2:**
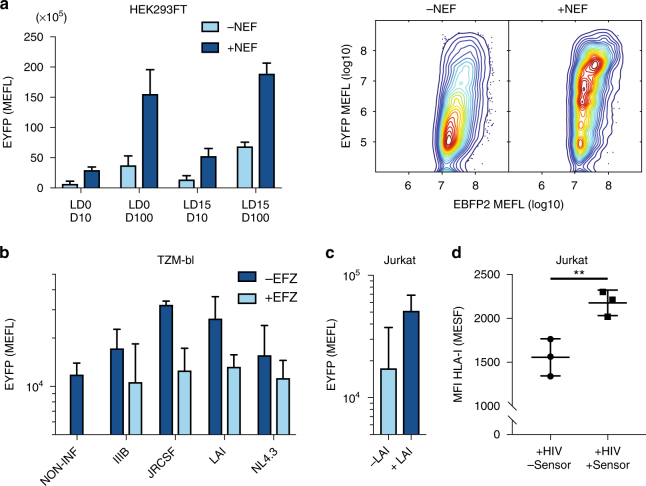
Nef-based HIV sensor-actuator. **a** Flow cytometry analysis of Nef devices (sdAb19-LD0/15-TCS(L)-GAL4-VP16, TEVp-LD0-SH3) in HEK293 cells treated with 10 nm or 100 nM Dox (D10, D100) 48 h post-transfection. Data show EYFP expression and standard deviation using molecules of equivalent fluorescein (MEFL) of EYFP for cells expressing >1 × 10^7^ MEFL of transfection marker Pacific-Blue. *n* = 2 independent technical replicates. Two-dimensional flow cytometry plots for the best variant (sdAb19-LD0/D10) are shown on the right. **b** Infection with HIV strains (IIIB, JRCSF, LAI, NL4.3) of TZM-bl cells expressing Nef device (sdAb19-LD0/D10). EYFP expression was compared to non-infected cells (NON-INF) and to cells infected and treated with the reverse transcriptase inhibitor Efavirenz (EFZ), showing that the device is sensitive to the inhibitory activity of the drug on viral genome retro-transcription. EYFP expression and standard deviation, using molecules of equivalent fluorescein (MEFL) of EYFP for cells expressing 2 × 10^5^ MEFL of transfection marker Pacific-Blue. *n* = 3 independent technical replicates. **c** Infection with HIV LAI strain of Jurkat cells expressing the Nef device (sdAb19-LD0/D10). EYFP expression and standard deviation using EYFP MEFL for cells expressing >1 × 10^5^ MEFL of transfection marker Pacific-Blue. *n* = 2 independent technical replicates. **d** HLA-I surface expression in Jurkat cells infected with LAI strain in the presence or absence of Nef device (sdAb19-LD0/D10). Immunostaining was followed by flow-cytometry of infected cells (gated on intracellular p24+ cells) expressing (+sensor) or not expressing (−sensor) the device. MFI indicates Mean Fluorescence Intensity. Results are mean ± s.e.m. of three independent experiments. Statistical significance was determined by a paired *t* test **p = 0.0046

Like Tat, Nef is an early stage gene expressed shortly after HIV infection^27,28^. Nef plays a prominent role in promoting viral infectivity, replication and evasion of host immune response by interfering with trafficking of several membrane receptors including CD4, CXCR4 and CCR5, as well as Human Leukocyte Antigen class I (HLA-I) molecules that present viral epitopes to CD8+ T cells^27,29^. Nef also facilitates infection of resting T cells and can induce a T cell activation program^30,31^. The Nef sensor device consists of a single domain antibody (sdAb19) that recognizes a conformational structure preserved in the core domain of the protein^32^ and SH3 which binds a proline-rich motif in Nef^33^. Both regions are conserved across different HIV strains. In contrast to our other sensors, SH3 is not an intrabody, but a domain of a p59^*fyn*^ protein tyrosine kinase that is highly expressed in T lymphocytes and implicated in antigen-induced T-cell activation^33^. The device exhibits up to 5-fold EYFP fold induction when co-transfected along with a Nef expressing plasmid in 293FT HEK and Jurkat T cells (Fig. 2a, Supplementary Fig 10).

### Nef sensor-actuator device detects several HIV strains

Next, we evaluated the ability to sense live HIV-1 infections in cells expressing the Nef-sensing device. Specifically, we performed infections with HIV X4 (NL4.3, LAI/IIIB) and R5 (JRCSF) strains that exhibit different co-receptor usage (CXCR4 or CCR5, respectively) into TZM-bl (a HeLa-based cell line). We observed EYFP expression upon HIV infection of TZM-bl cells, and also showed that when the infected cells were treated with reverse transcriptase inhibitor Efavirenz, reporter levels were near baseline (Fig. 2b, Supplementary Fig. 11). Jurkat T cells were then infected with the HIV LAI strain X4, confirming that the sensor device functions in T lymphocytes (Fig. 2c). The reduced response to HIV infection in both Jurkat and TZM-bl cells relative to plasmid expression of Nef in HEK cells may be due numerous reasons. For example, HIV-1 infected only a fraction of the cells (Supplementary Fig. 12), and in Jurkat cells the possible competition of p59^*fyn*^ with heterologous SH3 for binding to Nef may hamper reporter gene activation.

We then investigated whether our device interferes with Nef-mediated down-modulation of HLA-I in Jurkat cells and observed that indeed this function of Nef is impaired when the device is expressed (Fig. 2d). Importantly, the ability to sense an early gene in HIV infection may be useful for creating a potent programmable therapeutic agent that induces a localized CD8^+^ CTL response (Supplementary Note 4, Supplementary Fig. 13). Toward this goal we investigated whether a rewired Nef sensing device was able to induce selective expression of XCL-1 chemokine. XCL-1 is secreted by human CD8^+^ T cells along with other anti-HIV chemokines, exhibits a broad spectrum of activity against several biological variants of HIV-1, and interferes with infection at early stage of viral life cycle probably via direct interaction with the virus envelope^34^. We performed qPCR on 293FT HEK cells expressing Nef and observed that the device triggers transcription of XCL-1 in a selective manner (Supplementary Fig. 14).

## Discussion

We demonstrate a framework that uses intrabodies to connect sensing of intracellular proteins to chosen output activation. In this proof-of-concept study we tested the devices’ ability to respond to disease biomarker proteins for which intrabodies were previously developed. With technologies such as yeast and phage display libraries^35,36^, we can potentially obtain intrabodies against essentially any protein. Computational protein design tools such as Rosetta^37^ may be able to provide a large repertoire of synthetic sensing-actuator devices. Designing sensing modules toward conserved domains of the protein of interest may provide operation that is less sensitive to specific lineages and molecular evolution of the target (e.g., the Nef device operating across a broad spectrum of viral strains).

As is true for implementation of any sensor, its operation may impact the activity of the molecule that it is sensing. Therefore, an important future consideration is designing sensing modules that minimize or maximize interference with the activity of the protein of interest. To minimize interference, one may choose binding elements (e.g., intrabodies) that are less likely to obstruct the protein active site (although sequestration to the membrane could still lead to interference in some cases), or opt for intrabodies with different affinities^38,39^ toward the target to allow a broad dynamic range of interaction/interference. In other cases, it may be beneficial to inhibit proteins such as Nef, which induces HLA-I down modulation in T lymphocytes, and therefore one could choose binding elements (e.g., SH3) that impede binding with downstream or upstream partners. We envision that sensing HIV infected cells with a lentiviral-delivered Nef sensing-actuating genetic circuit may provide a means for studying the spatiotemporal characteristics of in vivo early viral infection. Such information could then help us design potent programmable therapeutic agents to induce a specific and localized CTL responses^34^ (Supplementary Note 4).

Intrabodies could also help create powerful tools to monitor diverse post-translational modifications (PTMs) in real time. PTMs not only serve as the most ubiquitous link in intracellular protein networks, but also play many other important roles, including in chromatin remodeling, transcription regulation, and cell signaling. Chirichella et al.^40^ have recently demonstrated newly designed intrabodies that bind acetylated proteins. By embedding this new class of intrabodies in our devices, our system can facilitate studies in a broad range of studies, e.g., ones that link PTMs to functional understanding epigenetic regulation or for spatiotemporally controlled transcriptional silencing. Our platform provides a strategy to translate aberrant protein expression, post-translational modification or viral infection into programmed transcriptional responses.

## Methods

### Cell culture

HEK293FT (Invitrogen) and HeLa-based TZM-bl (NIH AIDS reagent program) cells used in this study were maintained in Dulbecco’s modified Eagle medium (DMEM, Cellgro) supplemented with 10% FBS (Atlanta BIO), 1% penicillin/streptomycin/L-Glutamine (Sigma-Aldrich) and 1% non-essential amino acids (HyClone) at 37 °C and 5% CO_2_. Jurkat cells (ATCC) were maintained in RPMI-1640 (ATCC) supplemented with 10% FBS (Atlanta BIO), and 1% non-essential amino acids (HyClone) at 37 °C and 5% CO_2_. Doxycicline and Shield (Clonetech) were diluted according to manufacturer instructions.

### Transfection and fluorescence imaging

Protein sensor transfections were carried out in 24-well plate format. Transfections in HEK293FT were carried out with Attractene (Qiagen). A quantity of 300 ng of total DNA was mixed with DMEM base medium (Cellgro) without supplements to a final volume of 60 μl. A volume of 1.5 μl attractene was added to the dilutions and the samples were promptly vortexed to mix. The complexes were incubated for 20–25 min. During the incubation time, cells were harvested by trypsinization and 2 × 10^5^ cells seeded in 500 μl of complete culture medium in 24-well plate. Transfection complexes were added dropwise to the freshly seeded cells, followed by gentle mixing. Cells were supplemented with 1 ml of fresh growth medium 24 h posttransfection and analyzed by flow cytometry after 48 h. Co-localization imaging was performed after transfecting 293FT cells in 35 mm glass bottom dishes (Fluorodish). Cells were transfected with Lipofectamine LTX following manufacturer instructions. Total of up to 400 ng DNA was mixed with Opti-MEM I reduced serum medium (Life Technologies) to a final volume of 100 μl followed by addition of 0.5 μl PLUS reagent. After 5 min, 1.5 μl Lipofectamine LTX was added, the samples were briefly vortexed and incubated for 30 min at room temperature. During the incubation time, 2 × 10^5^ cells were harvested by trypsinization and seeded in 500 μl of complete culture medium in 24-well plate. Transfection complexes were added dropwise to the freshly seeded cells followed by gentle mixing. TZM-bl and Jurkat cells were electroporated with Neon Transfection System 10 μl Neon Tip (Life Technologies). For TZM-bl cells a total of 2 μg of DNA was prepared in a 1.5 mL tube. Meanwhile 2 × 10^5^ cells were harvested by trypsinization, and centrifuged in PBS at 150 g for 5 min at room temperature. Cells were resuspended in resuspension buffer R and then added to the DNA tube and gently mixed. The DNA and cell mixtures were picked with the appropriate Neon Tip and transferred to the electroporator and a pulse was applied (pulse voltage 1005 v, pulse width 35 ms, pulse number 2). 3 × 10^5^ Jurkat cells were resuspended in resuspension buffer R and electroporated with 4 μg of DNA prepared in a 1.5 ml tube as described above (pulse voltage 1325 v, pulse width 10 ms, pulse number 3). TZM-bl and Jurkat cells were infected with HIV strains around 6–12 h posttransfections, allowing for recovery after electroporation. Confocal imaging was performed with Leica TCS SP5 II microscope equipped with an incubation chamber using a ×63 objective. Other fluorescence and bright-field micrograph were acquired with Evos Cell Imaging System (Life Technology), using ×10 objective.

### Flow cytometry and data analysis

Cells were analyzed with LSR Fortessa flow cytometer, equipped with 405, 488, and 561 nm lasers (BD Biosciences). We collected 30,000–100,000 events per sample and fluorescence data were acquired with the following cytometer settings: 488 nm laser and 530/30 nm bandpass filter for EYFP/EGFP, 561 nm laser and 610/20 nm filter for mKate, and 405 nm laser, 450/50 filter for EBFP. Population of live cells was selected according to FCS/SSC parameters (Supplementary Fig. 20).

Flow cytometry data was converted from arbitrary units to compensated Molecules of Equivalent Fluorescein (MEFL) using the TASBE characterization method^41,42^. The TASBE method uses a strong constitutively expressed fluorophore, which serves as both a transfection marker and an indicator of relative circuit copy count. An affine compensation matrix is computed from single positive and blank controls. FITC measurements are calibrated to MEFL using SpheroTech RCP-30-5-A beads^41,43^ and mappings from other channels to equivalent FITC are computed from co-transfection of constitutively expressed EBFP, EYFP, and mKate, each controlled by the hEF1a promoter on its own otherwise identical plasmid. MEFL data are segmented by constitutive fluorescent protein expression into logarithmic bins at 10 bins/decade, and because the data are log-normally distributed, geometric mean, and variance computed for those data points in each bin. Based on the observed constitutive fluorescence distributions, a threshold was selected for each data set, below which data were excluded as being too close to the non-transfected population: 1 × 10^7^ MEFL for NS3 and NEF HEK, 3 × 10^7^ MEFL for HTT and TAT, 2 × 10^5^ MEFL for TZM-bl and 10^5^ for Jurkat data sets. High outliers are removed by excluding all bins without at least 100 data points. Both population and per-bin geometric statistics are computed over this filtered set of data. All experiments included at least three biological replicates and error bars indicate standard deviation. Variance for all groups is generally similar: any differences are reflected in the displayed standard deviation.

### HIV production and infection

HIV-1 strains were produced by transfection of HEK-293T cells with the corresponding infectious molecular clones (NIH AIDS reagents program) and JetPRIME® reagent (Polyplus transfection #114-07). After 40 h, virus preparations were concentrated by ultracentrifugation for 1 h, 64074 g, 4 °C on 20% sucrose to avoid viral particle-free proteins. Viral stocks were titered by HIV-1 p24 ELISA (PerkinElmer NEK050B001KT). For infection of TZM-bl cells, a viral inoculum of 500 ng of p24 was used for each strain. Fourty hours after infection, the cells were harvested, fixed, and permeabilized with cytofix/cytoperm solution (BD Biosciences #554722) for 15 min. The percentage of infected cells was then determined by intracellular staining of viral protein p24 with a conjugated antibody (KC57-FITC from Beckman Coulter #CO6604665, dilution 1:50) and flow-cytometry.

### HLA-I surface expression

Surface expression of HLA-I molecules was determined by staining before fixation with AlexaFluor® 647 mouse anti-human HLA-A, B, C antibody clone W6/32 (Biolegend® #311414, dilution 1:20). Fluorescence signals were then quantified with a flow-cytometer BD LSR-II system (Becton Dickinson) and FACSDiva8 software with the following settings: 640 nm laser and 670/14 nm filter. Flow cytometry data were converted from arbitrary units to compensated molecules of equivalent soluble fluorochrome (MESF) using Spherotech #RCP-30- 5A-2. Data were analyzed with FlowJo software. The mean of fluorescence (MFI) was determined and plotted for each condition. Results are from three independent experiments and error bars represent standard deviations.

### Apoptosis assays

Sensing-actuation devices transfections were performed along with pCMV-EGFP transfection marker. Sample cells including those in supernatant were collected 48 h posttransfection, washed with PBS, and stained with Pacific Blue conjugated 2.5 μL of Annexin V (LifeTechnologies) in 50 μL of binding buffer for 10 min at room temperature. Cells were analyzed by flow cytometry. Positive transfection cells (EGFP+) were gated and within this population we calculated apoptosis (cell death) induction, defined as the percentage of Pacific-Blue conjugated AnnexinV positive cells. All experiments included at least three biological replicates and error bars indicate standard error. Data analysis for the apoptotic assays was performed with FlowJo software.

### DNA cloning and plasmid construction

Plasmid vectors carrying gene cassettes were created using with Gateway system (Life technology), Infusion cloning system (Clonetech), or Golden Gate system (developed in-house). The list of all plasmids used in this study is shown in Supplementary Table 1.

### RNA extraction and RealTime PCR

RNA extraction was performed with RNeasy Mini Kit (Qiagen). Cells were washed in PBS and buffer RTL was added directly into the wells. Cells were then left there for 2 min, collected with a sterile scraper, and RNA was extracted according to manufacturer instructions. RNA was eluted in 30 μL of RNAse free water to maximize the yield. RNA samples were conserved at −80 °C. cDNA was obtained by using QuantiTect Reverse Transcription Kit (Qiagen) according to manufacturer’s instructions. The protocol was performed on ice in RNAse free environment to avoid RNA degradation. A negative control without Quantiscript Reverse Transcriptase was always prepared to assess contamination of genomic DNA of the RNA preparation. cDNA were diluted 1:10 and Fast SYBR Green Master Mix (ThermoFisher Scientific) was used to perform qPCR. Samples were loaded in MicroAmp™ Fast Optical 96-Well Reaction Plate (0.1 mL) and the experiment was carried out with a StepOnePlus™ 7500 Fast machine. Each well contained 20 μL of final volume (10 μL SYBR Green Master Mix, 7 μL ddHO, 1 μL of each primer, and 1 μL of template). Also, a control without template (blank) was set. 2^−ddCt^ was calculated to measure the fold change of output expression (XCL-1) in presence or absence of the target protein (Nef), after normalization of *C*_t_ values to endogenous housekeeping gene expression (GAPDH). Primers: GAPDH Forward GAAGATGGTGATGGGATTTC; GAPDH Reverse GAAGTTGAAGGTCGGAGT; XCL-1 Forward CTTGGCATCTGCTCTCTCACT; XCL-1 Reverse AGGCTCACACAGGTCCTCTTA.

### Data availability

The source data for all figures of this study as well as custom simulation code are available from the corresponding author upon reasonable request. Plasmid sequences have been deposited at GenBank under the accession codes found in Supplementary Table 1.

## Electronic supplementary material

Supplementary Information
