# Supplementary material for: Engineering modular intracellular protein sensor-actuator devices
Source: Nat Commun. 2018 May 14;9:1881. doi: 10.1038/s41467-018-03984-5 (PMC5951936; doi:10.1038/s41467-018-03984-5)
Supplement: Supplementary file 1 — Supplementary Information [file 41467_2018_3984_MOESM1_ESM.pdf]

Engineering modular intracellular protein sensor-actuator devices

Siciliano V. et al.

Supplementary Note 1: Tango/TEV mediated transcriptional regulation systems

TEVp is a protease widely used in proteomics to remove affinity tags after protein expression and purification, counteracting potential interference when performing structural and functional studies[1]. The ability to use TEV protease activity to release membrane bound transcription factors through the Tango/TEV technology has enabled the connection of extracellular inputs to transcriptional activation of chosen outputs[2–4]. In the pioneering work by Barnea et al., the GPCR receptor or IGF1R were adapted to detect the presence of an extracellular ligand (e.g. arginine vasopressin, AVP and IGF respectively)[2] (**Supplementary Fig. 2, (i)**). Binding of the ligand induces receptor phosphorylation and conformational change, resulting in recruitment of the Arrestin/Shc1-TEVp complex. TEVp then cleaves the cognate cleavage site on the GPCR/IGF1R fusion protein, releasing a transcription factor. Alternatively, estrogen receptors ERα and ERα/β (membrane tethered and fused to TEVp respectively) were used to induce reporter activation upon internalization of estrogen ligand which induces receptor dimerization and tTA release from the membrane (**Supplementary Fig. 2, (i)**) Similarly, a light-induced system (**Supplementary Fig. 2 (ii)**) operates on the principle of CRY2/C1BN dimerization from blue light excitation, which in turn releases the transcription factor via TEV protease cleaving the TEV cleavage site[4]. The MESA system (**Supplementary Fig. 2 (iii)**), uses a Tango/TEV-like architecture based on two trans-membrane protein chains to detect extracellular VEGF[3].

While all the aforementioned systems demonstrated sensing of extracellular inputs (ligands, light, protein), only the Tango estrogen-sensing system detects internalized ligand. In this system, two estrogen molecules bind independently to the two receptors (ERα and ERα/β), which results in ligand-induced hetero-dimerization and subsequent cleavage and release of the tethered transcription factor (**Supplementary Fig. 2 (i)** right). Our system, on the other hand, detects proteins by employing intracellular antibodies selected to bind distinct epitopes of the same target protein (**Supplementary Fig. 2 (iv)**). There is no requirement for any functional interaction between the target protein and the intrabodies that bind this protein. As such, our platform extends the Tango system to provide modular, rapid rewiring for targeting essentially any intracellular protein, which greatly enhances the utility of TEVp-based systems.

Supplementary Note 2: Actuation module characterization

A key element in the construction of sensing-actuation devices is to infer parameters that critically contribute to efficient sensing of the target proteins. To this end, we first designed and tested actuation modules that achieve sensitive and specific reporter activation. In order to obtain devices with significant ON/OFF ratio for output initiation, we designed actuation modules that include TEV cleavage sites with amino acid substitutions in P1' positions[5], resulting in altered affinity and cleavage activity of TEVp. Specifically, we included a canonical TCS (Serine in P1', **TCS-S,** *Km* 0.043; *Kcat* 0.194), or low affinity TCS (Leucine in P1', **TCS-L,** *Km* 0.240; *Kcat* 0.014) (see also **Supplementary Table 2**).

Intrabodies specific for our selected proteins were previously developed through techniques such as phage display[6], yeast display[7,8], or isolated from camelids[9] (**Supplementary Table 2**). For improved interactions between the two intrabodies and the target protein, and of TEVp with the TCS, we tested variants incorporating a flexible Glycine-Serine (G_4S) domain[10] (LD) of different lengths (0 Aa, 15 Aa). LDs were included between the intrabody/TCS and TEVp/intrabody fusion proteins.

We tested the ability of unbound non-localized TEVp to cleave TCS (S/L) by co-expressing in 293FT HEK cells hEF1α-TEVp along with membrane bound intrabody-TCS-GAL4-VP16 variants (**Supplementary Fig. 3a**) and GAL4-VP16 responsive fluorescent reporter (UAS-EYFP). TEVp induced significant activation of the reporter gene (up to 100 fold ON/OFF induction), indicating that the actuator module works in the outlined configuration (a trans-membrane component of the circuit and free cytosolic TEVp). Importantly, such a strong fold induction in presence of a constitutively expressed TEVp also suggests that careful tuning of protease expression is critical to maximize signal-to-noise ratio (**Supplementary Fig. 3b, c**).

Supplementary Note 3: Optimization of sensor-actuator devices for HTT and Tat proteins

The design and characterization of NS3 responsive device (**Supplementary Fig. 4)** served as initial analysis to identify the generalizable tuning parameters that enabled us to develop functional devices for other proteins of interest. For example, we inferred that fine-tuning of TEVp at the transcriptional level was critical for functional sensory devices, and different performance were obtained by varying linker domains, and having TEVp fused to the N- or C-terminus of the intrabody.

Intrabodies scFvs V_L12.3 and Happ1[11] used in the HTT sensor design are based on a previous study investigating the role of different domains in the toxicity of HTT protein. V_L12.3 binds the 17 N-terminal region of the first exon of HTT (HDx-1), while Happ1 binds a Proline-rich region of the same exon[11]. Similar to above, we created several device variants encoding TCS-S or TCS-L cleavage sites and different linker domains. We observed up to 4 fold induction in the presence of HTT-HDx-1 when TCS-S fused to Happ1 is co-expressed with N-terminal TEVp-V_L12.3 (with or without G-S linker domain), with Dox (0 or 10nM) (**Supplementary Fig. 7a-c**). We hypothesize that increased reporter fold induction with TCS-S may be due to epitope localization in HTT-HDx-1 that might not favor easy access of TEVp to the cleavage site, therefore requiring stronger affinity to induce reporter activation.

HIV-1 transactivator protein Tat is an indicator of HIV-1 infection. Tat is a key regulator of HIV replication, with localization that is mainly nuclear, although it has been shown to shuttle between the nucleus and cytoplasm depending on the stage of the infectious cycle[12]. We incorporated two scFvs (scFv2 and scFv3) fused to TEVp and TCS respectively and tested the sensor as previously described. We observed a 4.5 fold increase in EYFP fluorescence as a result of Tat sensing (**Supplementary Fig. 8a-c**).

Similar to the other sensors, the best performance for the HIV-1 Nef devices was obtained with a low affinity cleavage site (TCS-L), combined with TEVp fused to the N-terminus of SH3 and no linker domain. For this device, best results were obtained in the absence or at very low concentrations of Dox (D10).

Taken together, these data demonstrate that a critical element for tuning the sensor is TEVp activity, which can be modulated via transcriptional control and affinity of TEVp toward TCS. TCS-L exhibits better performance for the NS3, Tat and Nef devices, but for the HTT sensor, TCS-S is required to trigger output activation. Lastly, in all the devices, best output activation is obtained with TEVp at the N-terminal and without a flexible linker domain (although for Tat sensor, the TEVp at C/N-term did not affect device performance), suggesting perhaps that the chimeric protein structure facilitates access to TCS when complexed with target proteins.

Supplementary Note 4: Application of Nef sensing-actuation device to HIV

HIV represents a global health threat, with more than 30 millions people infected worldwide. The highly active anti-retroviral therapy (HAART) is able to reduce plasma virus levels, but after interruption in the therapy, HIV RNA levels become measurable in the plasma again[13]. The inability of HAART to completely eradicate the disease is likely due to the high mutation rate of the virus and existence of HIV-1 reservoirs in resting CD4+ T cells[14]. Nef protein plays a critical role in HIV-1 pathogenicity and viral replication. *In vitro* studies have shown the ability of Nef to downregulate CD4 and HLA-I, which prevents host immune cells from mounting an effective response against the virus[15] and to downregulate other proteins like SERCIN3/5, thereby enhancing viral replication.

The importance of HIV Nef protein early in viral pathogenesis and disease progression *in vivo* is clearly established[16]. It has been reported that during HIV-1 infection, unintegrated form of viral cDNA is detectable *in vivo*, and that the unintegrated DNA results in translation of early viral genes such as Nef, which downregulates CD4, CXCR4 and CCR5 receptors[17]. Quiescent T cells infected with HIV-1 NL4-3 strain showed substantial expression of *nef* transcript and protein expression[18], and infection of resting T cells with Nef-negative HIV-1 resulted in diminished T-cell sensitization to activation by CD3-CD28 beads as compared to wild-type virus[18]. This evidence supports the hypothesis that expression of the earliest HIV genes may be needed to increase T cell activity in quiescent T cells, indispensable for viral replication and spreading. Sensing and targeting of HIV infected cells with a lentiviral-delivered Nef sensing-actuating genetic circuit may provide useful spatiotemporal mapping of *in vivo* early viral infection. It may also act as a potent programmable therapeutic agent to induce a specific and localized CTL response[19] via release of therapeutic chemokines encoded by our device in HIV-1 infected CD4+T cells. For example, by creating a therapeutic variant of the device, secretion of chemokines such as XCL-1/lymphotactin, might help recruit to the site of infection XCR1+ immune cells such as CD8+ T cells[20] and cross-presenting Denditric Cells (DC) able to induce potent CD8+ T-cell responses as already demonstrated for influenza[21] or T-cell therapy of tumors[19,21–23]. Furthermore, XCL1 would inhibit HIV entry into surrounding CD4+ T cells by binding directly to HIV envelope protein[24,25]. A schematic illustration of the system in shown in **Supplemental Figure 13.**

We rewired Nef sensing device to activate XCL-1 expression in Nef expressing cells. We measured transcriptional activation upon Nef sensing by qPCR and show that the device is able to trigger chemokine output (**Supplementary Fig.14**), opening new opportunities for immunotherapies. XCL1 was also found in higher concentrations in elite controllers (ECs), a subset of HIV infected individuals that maintain undetectable viral replication in the absence of anti-retroviral therapy, in comparison to XCl-1 levels in non-controllers or uninfected individuals[26]. Globally, our device could enable cell-mediated localized immunotherapy in tissues that are otherwise not accessible to conventional antiviral therapy, such as the gut[27].

Supplementary Note 5: Computational model for protein-sensor design principles

To investigate the dynamics of the protein sensor topology and to uncover design principles to guide further engineering, we implemented and analyzed a simple computational model of the platform. A system of ordinary, first-order differential equations (ODEs) describing potential interactions of species involved in the protein sensor was constructed and solved using MATLAB[28].

The model includes 15 molecular species (see **Supplementary Fig. 15a** and descriptions below), which interact through their various domains: the antibody (Ab) domains of both the membrane-bound sensor part (*M*) and the cytosolic sensor part (*E*) can bind the protein of interest (*P*) reversibly with the same forward and reverse rates, k_{1f} and k_{1r} respectively and dissociation constant K_D:

$$K_D = \frac{k_{1r}}{k_{1f}}$$

The TEV protease domain of *E* can bind the TEV cleavage site (TCS) of *M* reversibly with forward and reverse rates, k_{2f} and k_{2r} respectively, followed by irreversible cleavage of the TCS with rate k_{CAT}, releasing the transcription factor, *T*. These rates can be summarized by the Michaelis constant, K_M:

$$K_M = \frac{k_{2r} + k_{CAT}}{k_{2f}}$$

Reactions are shown in **Supplementary Fig 15b**. We assume that when the two parts of the sensor are bound at either the TEV protease cleavage site (TCS) end or the antibody (Ab) end, subsequent binding at the unbound end is enhanced due to increased local effective concentration factor (*CF*) as described previously[29].

Each simulation was initialized with concentration values (*C0*) for each sensor part (*M* and *E*). Fold change was calculated at t=24 hours by taking the concentration of cleaved transcription factor product, *T*, for a simulation initialized with the protein of interest, *P*, divided by the concentration of *T* from a corresponding simulation with P=0 (see **Supplementary Fig. 16**).

To understand how the sensor is affected by the kinetics of the antibody domains and TEV protease domains as well as molecular concentrations, we simulated various kinetic rate constants and initial concentrations (see **Supplementary Table 3**). The sensor performs better for increased values of binding enhancement, CF, after binding at one part of the sensor (**Supplementary Fig. 17**) and sets an upper limit for achievable fold change. Since the CF factor greatly confounds the results, in subsequent simulations its value was fixed. We also find improved sensor performance when the antibodies bind the protein of interest much stronger than TEV protease binds the TCS, which occurs at lower values of K_D and higher values of K_M (**Supplementary Fig. 18a**). Intuitively, this allows for sensor complex to form only in the presence of the protein and also decreases the rate of cleavage in the case of off-target TEV:TCS binding in the absence of protein. This is in congruence with our experimental results where we find that the TCS with increased KM

and slower k_{CAT} (TCS-L in **Supplementary Table 2**) improves sensor performance (see **Supplementary Fig. 4b** and **Supplementary Fig. 4c**). The fold change also increases for lower sensor concentrations and for optimal concentrations of protein of interest (**Supplementary Fig. 18b**). Our experimental results, which show that lower levels of doxycycline (i.e. lower levels of sensor production) perform best, corroborate this finding. In practice, there is likely a lower limit for k_{CAT} and sensor concentration (*C0*) and an upper limit for K_M due to background fluorescence. Note that the non-monotonic response to protein of interest is not surprising due to the bipartite nature of the system. Such behavior has been previously described for reactions with similar topology such as receptor cross-linking[30] and supports the experimental results in **Supplementary Figure 5a**.

Overall, from these simulations we can draw five main conclusions, which we believe will be valuable in designing new protein sensors based on our platform (summarized in **Supplementary Fig. 19**):

1. Careful design of sensor parts is necessary to ensure that binding the protein of interest by both parts greatly enhances binding of TEV to the TCS (i.e. CF is as high as possible).
2. Sensor performance improves when the antibodies bind the protein of interest strongly.
3. Sensor performance generally improves for weaker TEV protease binding and slower TEV protease cleavage rate (k_{CAT}). This binding should be much weaker than binding of the antibodies to the protein of interest.
4. To a certain extent, lower sensor concentrations reduce off-target cleavage and therefore improve sensor performance
5. The sensor's response to protein of interest levels is not monotonic: while likely not physiologically relevant, too much protein can saturate the sensor.

Species (see Supplementary Fig. 15a):

M	Membrane-bound sensor part 1
E	Cytosolic sensor part 2
P	Protein of interest (POI)
m	Cleaved sensor part 1
T	Cleaved transcription factor (product)
MP	Sensor part 1 bound to POI
EP	Sensor part 2 bound to POI
mP	Cleaved sensor part 1 bound to POI
ME	Sensor part 1 bound to sensor part 2 through TCS
MPE	Sensor part 1 bound to sensor part 2 through POI
M2EP	Sensor part 1 bound to POI-bound sensor part 2 through TCS
MP2E	POI-bound sensor part 1 bound to sensor part 2 through TCS
MPEP	POI-bound sensor part 1 bound to POI-bound sensor part 2 through TCS
MPE2	Sensor part 1 bound to sensor part 2 through both TCS and POI
mPE	Cleaved sensor part 1 bound to sensor part 2 through POI

Reactions:

$$\frac{dM}{dt} = -k1f \cdot M \cdot P + k1r \cdot MP - k1f \cdot M \cdot EP + k1r \cdot MPE - k2f \cdot M \cdot E + k2r \cdot ME - k2f \cdot M \cdot EP + k2r \cdot M2EP$$

$$\frac{dE}{dt} = -k1f \cdot E \cdot P + k1r \cdot EP - k1f \cdot E \cdot MP + k1r \cdot MPE - k2f \cdot E \cdot M + k2r \cdot ME - k2f \cdot E \cdot MP + k2r \cdot MP2E + kCAT \cdot ME + kCAT \cdot MP2E$$

$$\frac{dP}{dt} = -k1f \cdot M \cdot P + k1r \cdot MP - k1f \cdot E \cdot P + k1r \cdot EP - k1f \cdot m \cdot P + k1r \cdot mP - k1f \cdot ME \cdot P + k1r \cdot M2EP - k1f \cdot ME \cdot P + k1r \cdot MP2E - k1f \cdot M2EP \cdot P + k1r \cdot MPEP - k1f \cdot MP2E \cdot P + k1r \cdot MPEP$$

$$\frac{dm}{dt} = -k1f \cdot m \cdot P + k1r \cdot mP - k1f \cdot m \cdot EP + k1r \cdot mPE + kCAT \cdot ME + kCAT \cdot M2EP$$

$$\frac{dT}{dt} = kCAT \cdot ME + kCAT \cdot M2EP + kCAT \cdot MP2E + kCAT \cdot MPEP + kCAT \cdot MPE2$$

$$\frac{dMP}{dt} = k1f \cdot M \cdot P - k1r \cdot MP - k1f \cdot MP \cdot E + k1r \cdot MPE - k2f \cdot MP \cdot E + k2r \cdot MP2E - k2f \cdot MP \cdot EP + k2r \cdot MPEP$$

$$\frac{dEP}{dt} = k1f \cdot E \cdot P - k1r \cdot EP - k1f \cdot EP \cdot M + k1r \cdot MPE - k1f \cdot EP \cdot m + k1r \cdot mPE - k2f \cdot EP \cdot M + k2r \cdot M2EP - k2f \cdot EP \cdot MP + k2r \cdot MPEP + kCAT \cdot M2EP + kCAT \cdot MPEP$$

$$\frac{dmP}{dt} = k1f \cdot m \cdot P - k1r \cdot mP - k1f \cdot mP \cdot E + k1r \cdot mPE + kCAT \cdot MP2E + kCAT \cdot MPEP$$

$$\frac{dME}{dt} = k2f \cdot M \cdot E - k2r \cdot ME - k1f \cdot ME \cdot P + k1r \cdot M2EP - k1f \cdot ME \cdot P + k1r \cdot MP2E - kCAT \cdot ME$$

$$\frac{dMPE}{dt} = k1f \cdot M \cdot EP - k1r \cdot MPE + k1f \cdot MP \cdot E - k1r \cdot MPE - k2f \cdot CF \cdot MPE + k2r \cdot MPE2$$

$$\frac{dM2EP}{dt} = k1f \cdot ME \cdot P - k1r \cdot M2EP + k2f \cdot M \cdot EP - k2r \cdot M2EP - k1f \cdot CF \cdot M2EP + k1r \cdot MPE2 - k1f \cdot M2EP \cdot P + k1r \cdot MPEP - kCAT \cdot M2EP$$

$$\frac{dMP2E}{dt} = k1f \cdot ME \cdot P - k1r \cdot MP2E + k2f \cdot MP \cdot E - k2r \cdot MP2E - k1f \cdot CF \cdot MP2E + k1r \cdot MPE2 - k1f \cdot MP2E \cdot P + k1r \cdot MPEP - kCAT \cdot MP2E$$

$$\frac{dMPEP}{dt} = k1f \cdot M2EP \cdot P - k1r \cdot MPEP + k1f \cdot MP2E \cdot P - k1r \cdot MPEP + k2f \cdot MP \cdot EP - k2r \cdot MPEP - kCAT \cdot MPEP$$

$$\frac{dMPE2}{dt} = k1f \cdot CF \cdot MP2E - k1r \cdot MPE2 + k1f \cdot CF \cdot M2EP - k1r \cdot MPE2 + k2f \cdot CF \cdot MPE - k2r \cdot MPE2 - kCAT \cdot MPE2$$

$$\frac{dmPE}{dt} = kCAT \cdot MPE2 + k1f \cdot m \cdot EP - k1r \cdot mPE + k1f \cdot mP \cdot E - k1r \cdot mPE$$

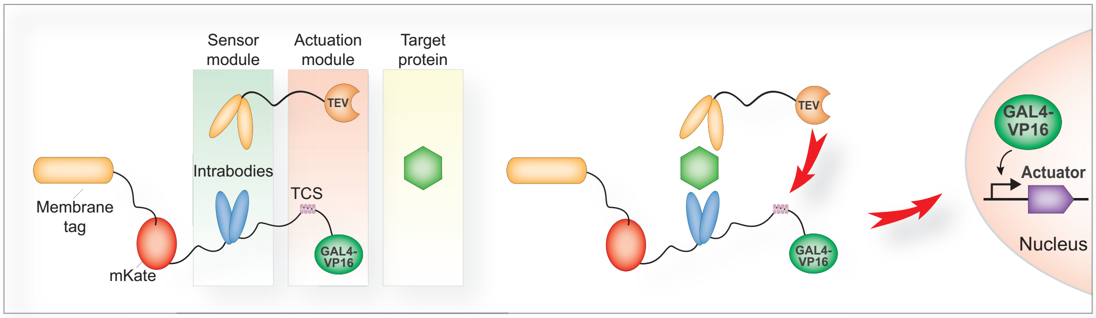

Supplementary Figure 1: Sensing-actuation genetic device. Sensing and actuation is performed through interactions between the target protein and two chimeric proteins. The sensing module comprises intracellular antibodies. The actuation module consists of the Tobacco Etch Virus protease (TEVp) coupled to GAL4-VP16 transcriptional activator. The device integrates sensing and actuation modules with protein biomarker detection similar to a 3-input AND Boolean logic gate, since all three components are needed in order to trigger output expression. Note that fine tuning of component levels and interaction strength is needed to achieve correct behavior where output expression increases as a function of target protein levels.

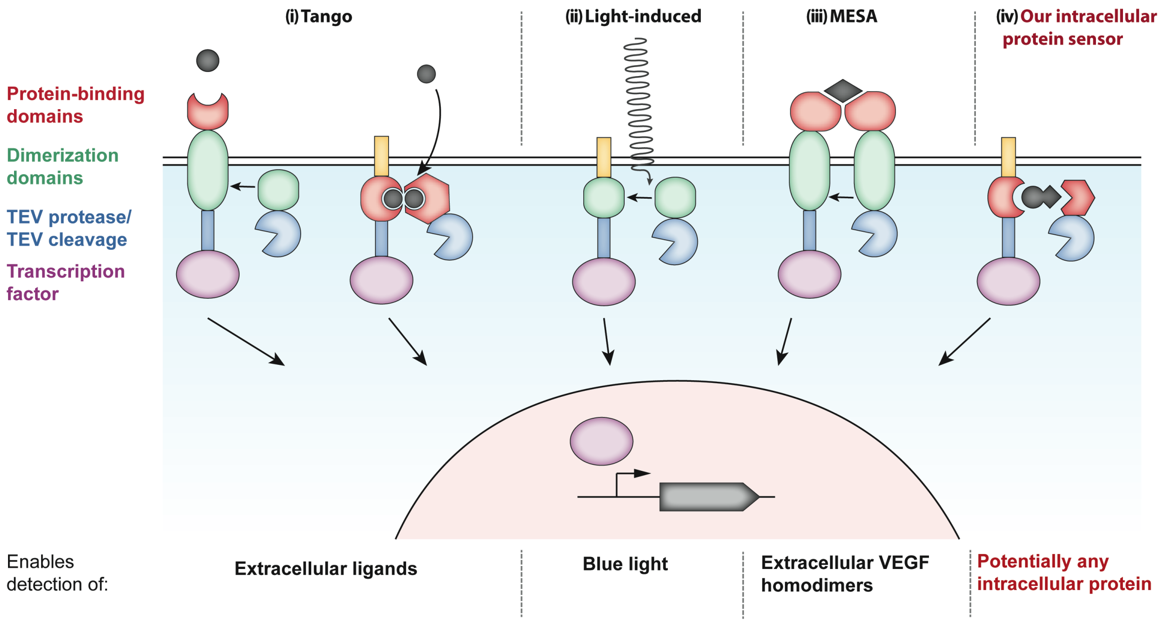

Supplementary Figure 2: TEVp-based sensor systems. From the left: i) Tango/TEV technology has been demonstrated to connect extracellular inputs to output activation. Tango assays used to monitor the activity of three different classes of receptors (i): (left) G protein-coupled receptors AVPR2, receptor tyrosine kinases (IGFR1), and (right) estrogen hormone receptors[2]. Binding of extracellular ligand to AVPR2 or IGFR1 results in receptor phosphorylation and recruitment of partner proteins fused to TEVp (i), while Tango estrogen-sensing system detects internalized ligand. The latter detection system operates through the use of receptors that dimerize after each binding the estrogen ligand. ii) Blue Light-induced CRY2/C1BN dimerization releases the transcription factor via TEV protease cleavage of the cognate site[4]; iii) MESA system relies on two trans-membrane protein chains linked to TEV to detect extracellular VEGF homodimer[3]; iv) our intracellular protein sensing actuation devices is based on modular intracellular antibodies that bind to distinct epitopes of the same protein of interest for TEVp-mediated transcription factor membrane. Our device therefore extends the previously described Tango platform to potentially detect the presence of any protein for which we can find or engineer intracellular antibodies for two different epitopes.

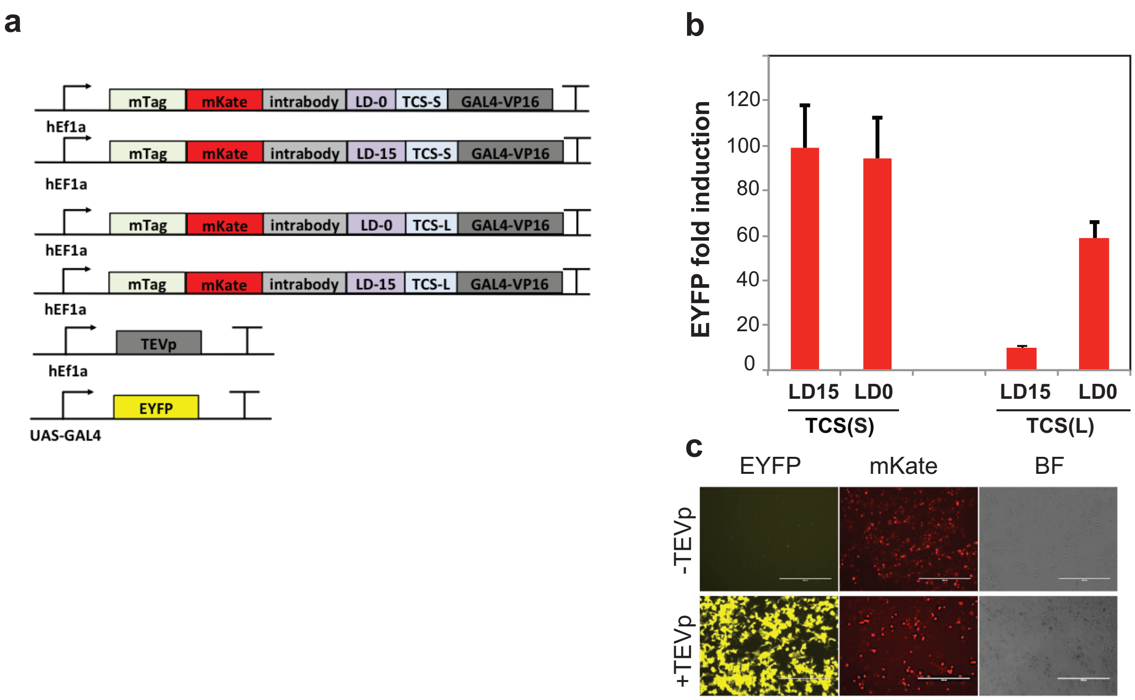

Supplementary Figure 3: Actuation module characterization. **(a)** Variants of the protein sensing architecture. mTag is the membrane anchor, mKate is the red fluorescent protein, the intrabody used in this set of experiment is scFv35 (binding NS3). TCS of different affinities toward TEVp (TCS-S/L) include flexible Glycine-Serine (G4-S) linker domains (LD0/LD15) were tested. The modules are expressed from hEf1α promoter. **(b)** Membrane-anchored variants were co-transfected in 293FT HEK cells along with GAL4-VP16 responsive promoter activating EYFP reporter gene, with or without hEF1α-TEVp. EYFP was measured 48 hours post transfection by flow cytometry. Bars show EYFP fold induction and standard deviation of +/- TEVp for cells expressing >1×10^{7} MEFL of transfection marker mKate for n=3 replicates. **(c)** Representative fluorescence microscopy images show EYFP activation in cells expressing TEVp for LD0-TCS-L variant in the presence of constitutively expressed TEVp. Scale bars indicate 200μm.

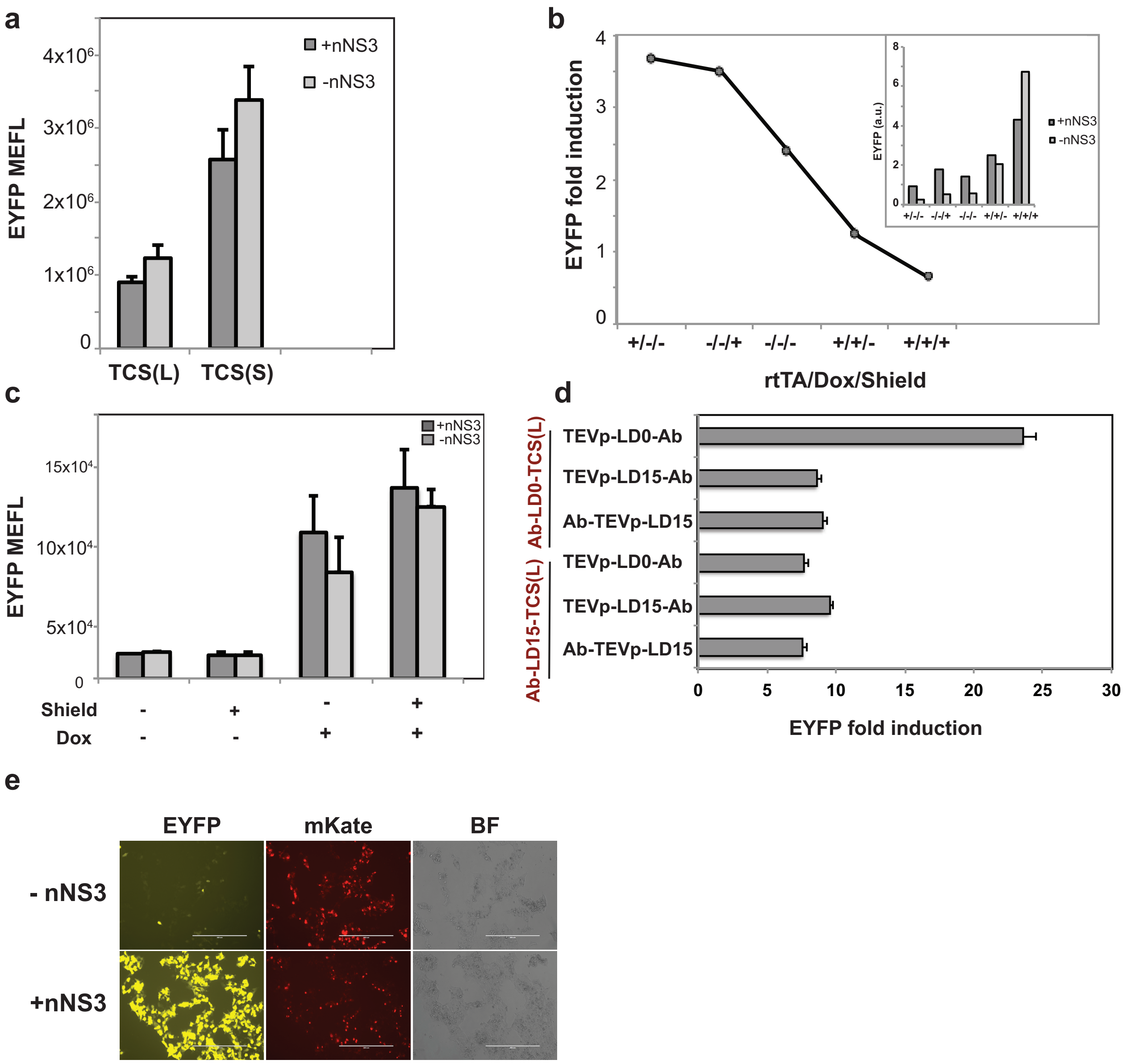

Supplementary Figure 4: NS3 sensor characterization. **(a)** Membrane tethered LD15-TCS-S/L variants of nNS3 sensor were co-expressed with hEF1a-TEVp-scFv162 in the presence or absence of nNS3 in 293FT HEK cells. For these first attempts we observed EYFP activation regardless of nNS3 expression, indicating that fine-tuned regulation of protease activity is critical for sensitive and specific activation of reporter genes. Data were collected 48h post-transfection and represent geometric mean and standard deviation of means of EYFP MEFL for cells expressing $>1\times10^7$ MEFL of transfection marker mKate for n=3 technical replicates. **(b)** TEVp fused at the C-terminal of scFv162 (DD-scFv162-LD15-TEVp, see **Supplementary Table 1**) regulated by Dox and Shield was assayed with LD15-TCS(L) variant of the genetic device. EYFP geometric mean was measured in the

absence or presence of nNS3 after treatment with Dox (0-1000nM), Shield (0-1000 nM) or both, also in the presence or absence of the transcriptional activator rtTA3 expressed constitutively by hEF1a promoter. Data show near 4x nNS3 induced EYFP activation with basal TEVp expression, either in the presence of rtTA3 only or shield only (which stabilizes TEVp due to DD domain) suggesting that the sensor works well with low levels of TEVp. EYFP fold activation is generally inversely related to TEVp levels for the conditions tested. **(c)** We performed a similar experiment to (b), but using membrane anchored, high affinity LD15-TCS(S). EYFP geometric mean was measured in the absence or presence of NS3 after treatment with Dox, Shield or both (as above). High levels of EYFP were detected 48h post-transfection in the presence of Dox even when nNS3 was not expressed, demonstrating that even basal levels of TEVp have significant interaction with TCS(S). **(d)** Flow cytometry analysis of six device variants including C-N terminal fusions of TEVp-intrabody, linker domains and low affinity TCS(L). The devices were tested in the presence and absence of nNS3. Data show EYFP fold induction and standard deviation of fold induction using molecules of equivalent fluorescein (MEFL) of EYFP for cells expressing $>1\times10^7$ MEFL of transfection marker mKate. n=3 independent technical replicates. Ab indicates scFv35 intrabody for TCS fusions and scFv162 intrabody for TEVp fusions. **(e)** Representative fluorescence micrographs of the best nNS3 sensing-actuation device (scFv35-LD0-TCS(L)/TEVp-LD0-scFv162). Scale bar is 200μm.

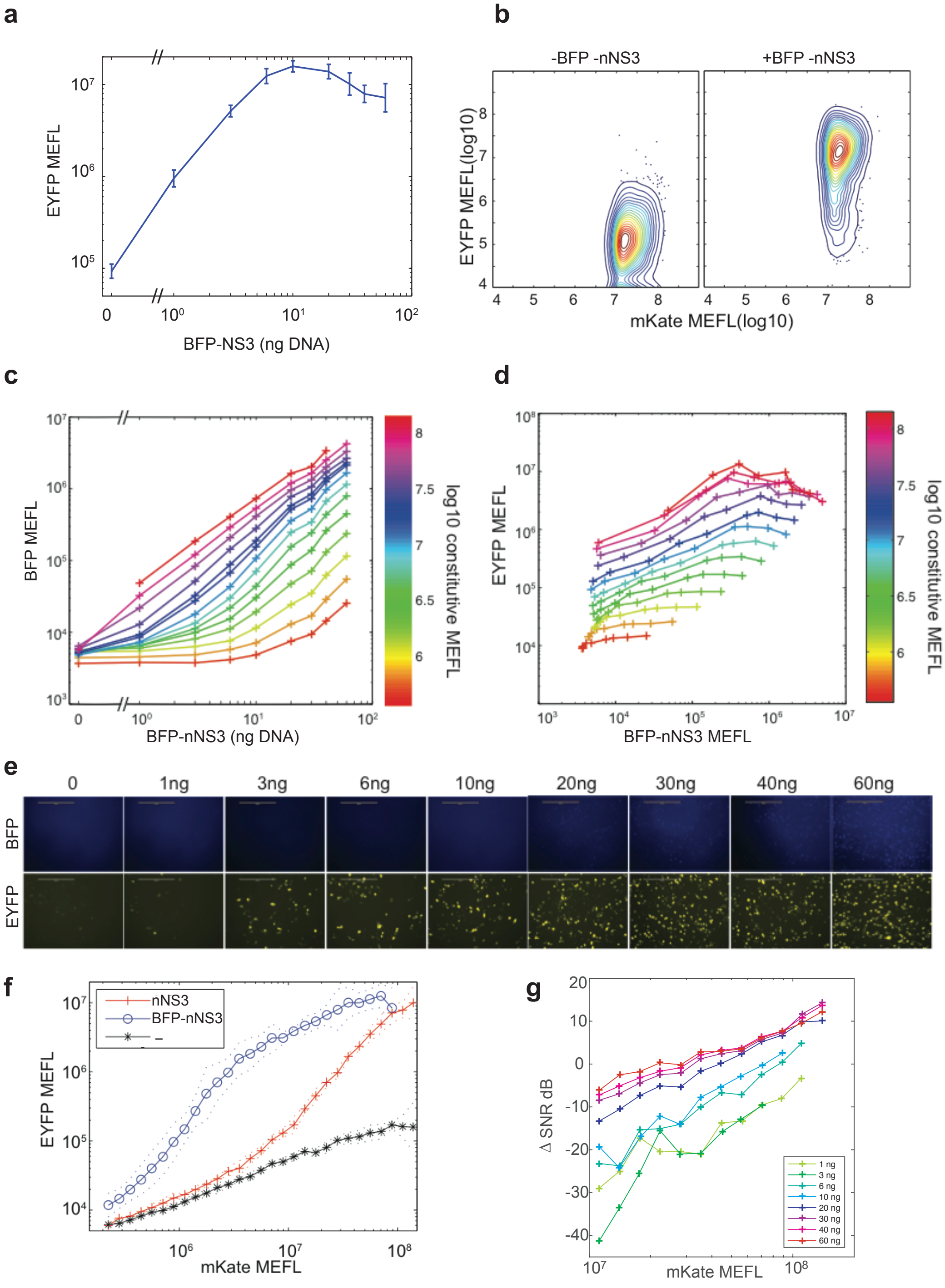

Supplementary Figure 5: BFP-nNS3 sensing characterisation. **(a)** We co-transfected 293FT HEK cells with the best nNS3 sensory device (scFv35-LD0-TCS(L)/TEVp-LD0-scFv162, Fig. 1c, Supplementary Fig. 4d) and different concentrations of BFP-nNS3 encoding plasmid. **(b)** Representative two-dimensional flow cytometry plots for the scFv35-LD0-TCS(L)/TEVp-LD0-scFv162 variants. **(c)** Relationship between BFP-nNS3 dosage and BFP. We used constitutive mKate marker to separate cells into subpopulations by relative transfection dose, segmenting at 10 bins per decade of mKate

MEFL. Within each subpopulation, BFP fluorescence increases in approximately linear fashion with BFP-NS3 plasmid dose once expression is significantly above auto-fluorescence. **(d)** Relationship between BFP-nNS3 MEFL and EYFP output. For each binned BFP subpopulation from (c) we estimated EYFP fluorescence levels. **(e)** Representative fluorescence micrographs depicting EYFP fluorescence as a function of increasing concentration of BFP-nNS3 plasmid transfection. Scale bar 200μm. **(f)** Comparison of EYFP levels induced by BFP-nNS3, nNS3 and a negative control comprising the device (scFv35-LD0-TCS(L)/TEVp-LD0-scFv162) in absence of the target protein (black line) as a function of transfection efficiency. Constitutive mKate expression was used as indicator of relative circuit copy count. Data represent geometric mean and standard deviation of means of EYFP MEFL for cells expressing >1 × 10^7 MEFL of transfection marker mKate for n=3 technical replicates. **(g)** Relation between transfection level, BFP-nNS3 dosage and detection sensitivity: difference in signal-to-noise ratio between input (BFP-nNS3) and output (EYFP) signals, calculated with respect to transfection level marker (mKate) per[31], for each positive dosage using 0 ng BFP-nNS3 as the negative condition. Response is undetectable below 10 ng dosage, but for 20 ng and above high levels of transfection have significantly positive ΔSNR.

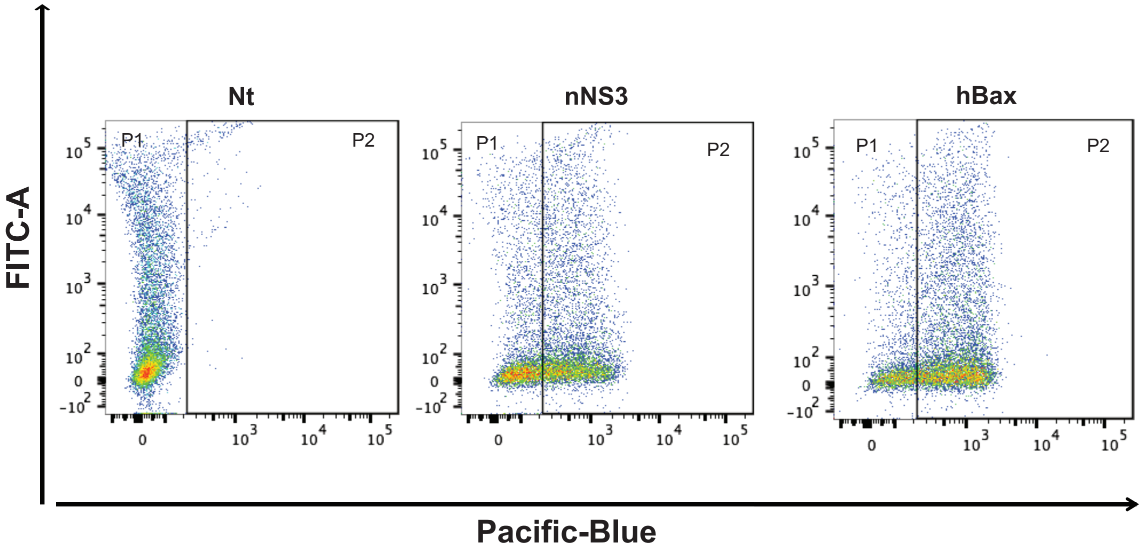

Supplementary Figure 6: Representative two-dimensional flow cytometry plots for apoptotic assay in 293FT cell culture with nNS3 sensor device, using the pro-apoptotic gene hBax as output (**Fig. 1d**). 293FT cells were co-transfected with the genetic device (scFv35-LD0-TCS(L)/TEVp-LD0-scFv162/UAS-hBax) alone (Nt) or along with nNS3, or CMV-hBax (hBax). Pacific-Blue conjugated to AnnexinV staining and flow cytometry were performed 48h post-transfection.

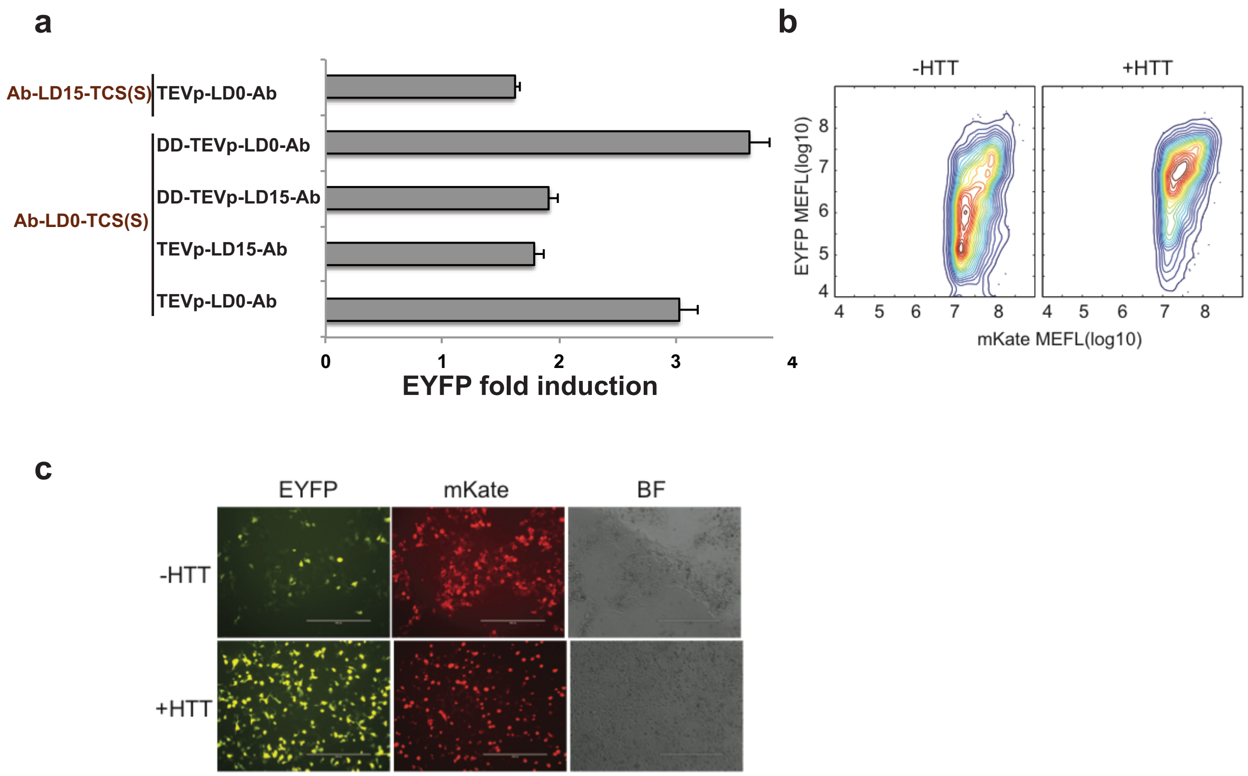

Supplementary Figure 7: HTT sensor characterization. **(a)** Flow cytometry analysis of device variants in the presence and absence of HTT performed 48h post-transfection. Ab indicates Happ1 intrabody for TCS fusions and VI12.1 intrabody for TEVp fusions. Data shows EYFP fold induction and standard deviation of EYFP MEFL for cells expressing >1×10^7 MEFL of transfection marker mKate. n=3 independent technical replicates. **(b)** Representative two-dimensional flow cytometry plots for Happ1-LD0-TCS(S)/DD-TEVp-LD0-VI12.1, with and without HTT. **(c)** Representative fluorescence micrographs of EYFP activation upon HTT sensing, Happ1-LD0-TCS(S)/DD-TEVp-LD0-VI12.1 variants. BF: bright field. Scale bar 200μm.

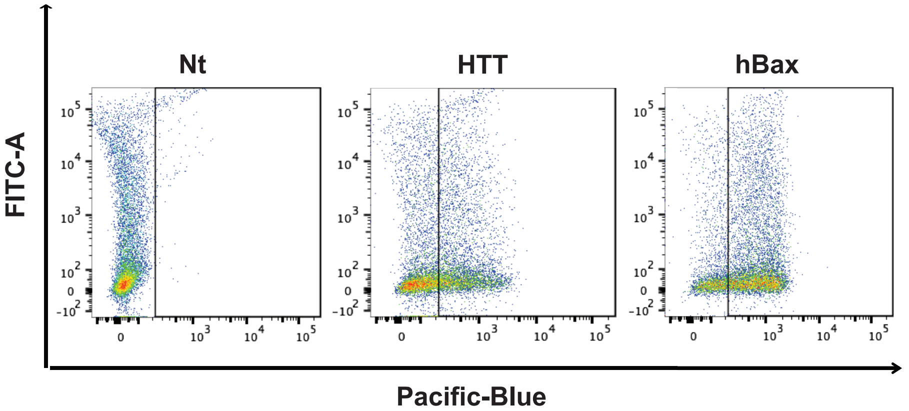

Supplementary Figure 8: Representative two-dimensional flow cytometry plots for apoptotic assay in 293FT HEK cell culture with HTT device with pro-apoptotic gene hBax as output (**Fig. 1f**). 293FT cells were co-transfected in absence of the target protein (Nt), HTT (Happ1-LD0-TCS(S)/DD-TEVp-LD0-VI12.1/UAS-hBax) or with CMV-hBax (hBax). Pacific Blue AnnexinV staining and flow cytometry were performed 24h post-transfection.

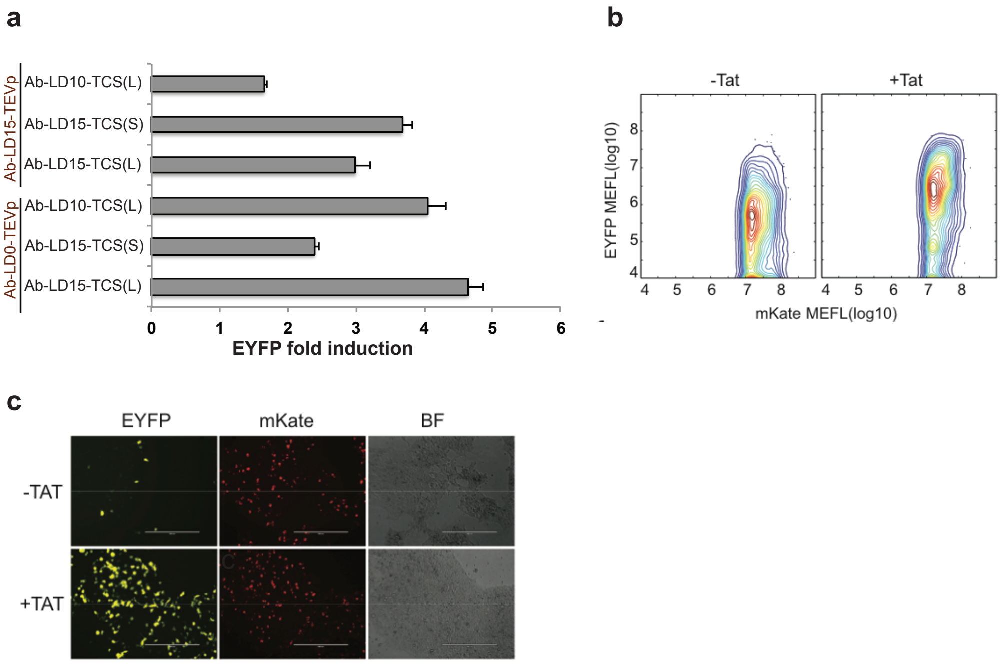

Supplementary Figure 9: Tat sensor characterization. **(a)** Flow cytometry analysis of device variants in the presence and absence of Tat performed 48h post-transfection in 293FT HEK cells. Ab indicates scFv3 intrabody for TCS infusions and scFv2 intrabody for TEVp infusions. Data shows EYFP fold induction and standard deviation of EYFP MEFL for cells expressing $>1\times10^7$ MEFL of transfection marker mKate. n=3 independent technical replicates. **(b)** Representative two-dimensional flow cytometry for scFv3-LD15-TCS(L)/scFv2-LD0-TEVp, with or without Tat. **(c)** Fluorescence micrographs of EYFP activation upon Tat sensing for scFv3-LD15-TCS(L)/scFv2-LD0-TEVp variants. BF: bright field. Scale bar 200μm.

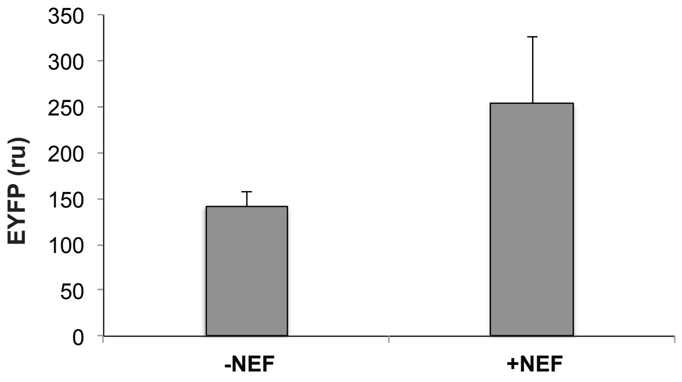

Supplementary Figure 10: Nef device response in Jurkat cells. Best device variant (sdAb19-LD0/D10) (Fig. 2a) was transfected in Jurkat cells in presence or absence of Nef. Data show EYFP expression and standard deviation using EYFP MEFL for cells expressing >1×10^5 MEFL of transfection marker Pacific-Blue. n=2 independent technical replicates.

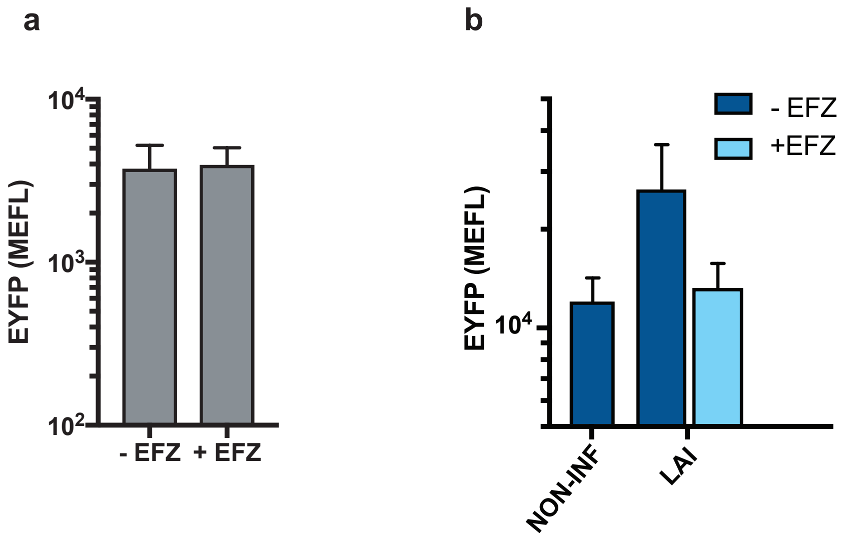

Supplementary Figure 11: Nef device response to Efavirenz treatment in non-infected TZM-bl cells (**Fig. 2b**). TZM-bl cells were transfected with Nef device (LD0-TCS(L)/TEVp-LD0-SH3) and treated with the reverse transcriptase inhibitor Efavirenz (EFZ). **(a)** Data show that Efavirenz treatment does not result in EYFP activation in absence of HIV infection. **(b)** Infection with HIV LAI strain of TZM-bl cells expressing Nef device (sdAb19-LD0/D10) and comparison of EYFP expression levels in non-infected cells (NON-INF) and in cells treated with the reverse transcriptase inhibitor Efavirenz (EFZ). Data indicate that the device is sensitive to the inhibitory activity of the drug on viral genome retro-transcription. EYFP expression and standard deviation using molecules of equivalent fluorescein (MEFL) of EYFP for cells expressing $>2\times10^5$ MEFL of transfection marker Pacific-Blue. n=2 independent technical replicates.

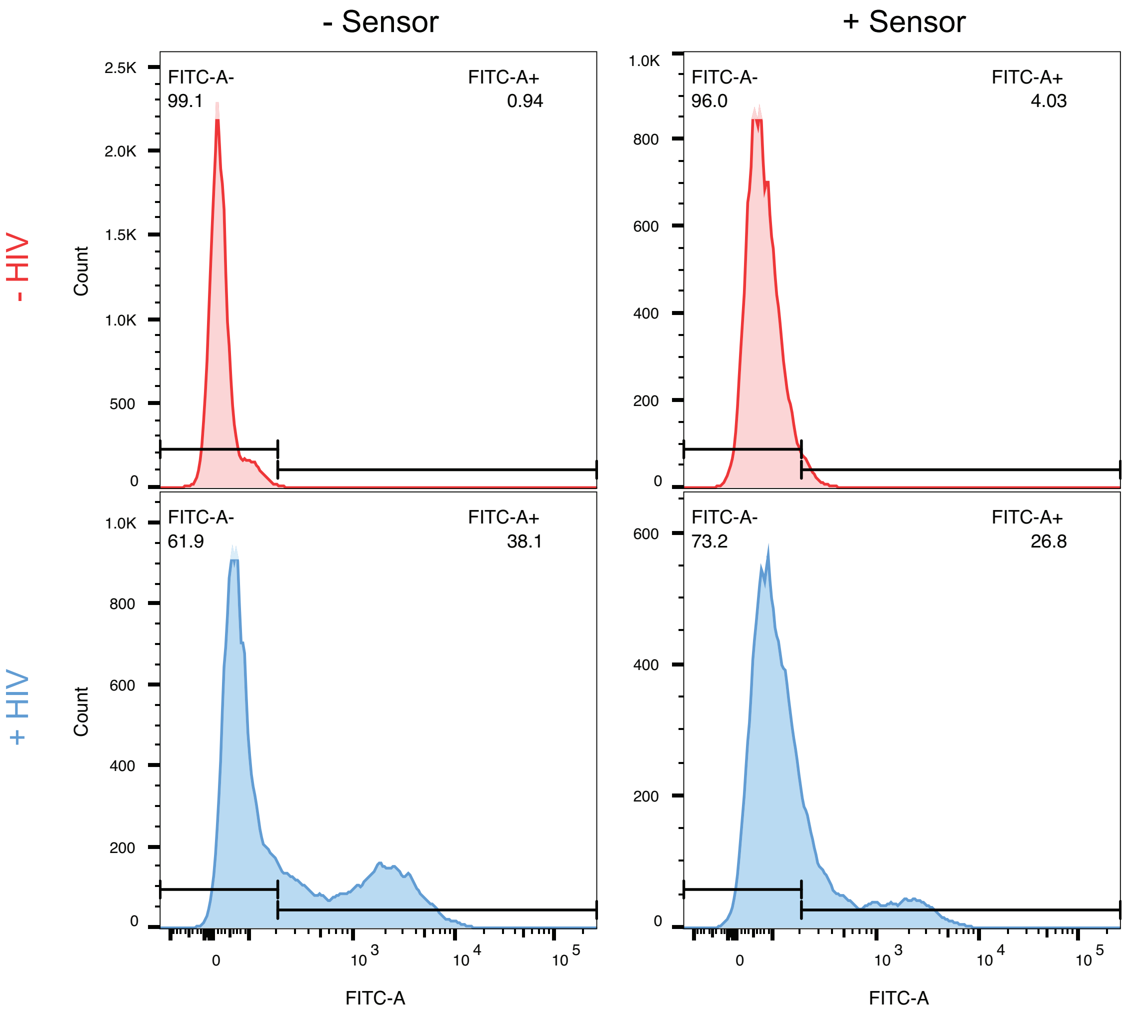

Supplementary Figure 12: p24 staining of HIV-1 infected Jurkat T cells. The percentage of infected cells was determined by intracellular staining of viral protein p24 with a conjugated antibody and flow-cytometry. The presence of the sensor does not reduce p24 staining.

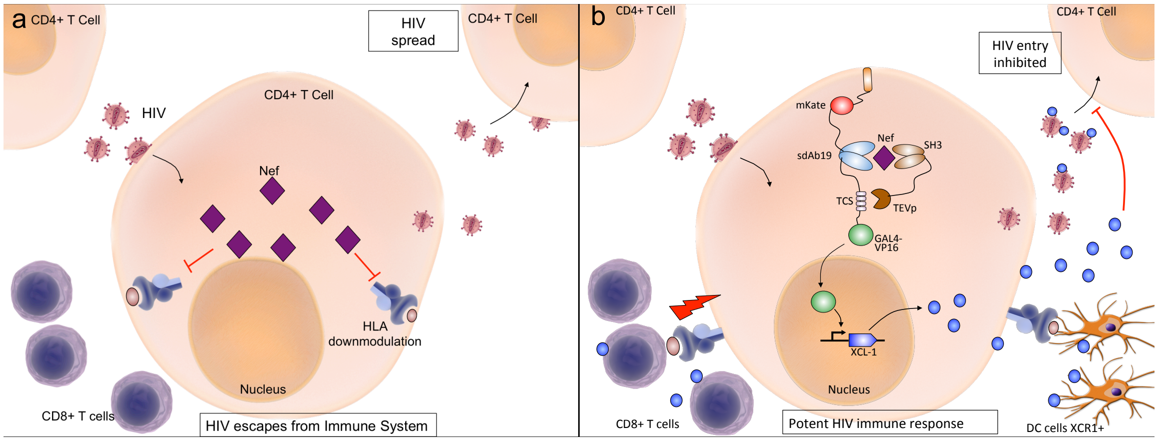

Supplementary Figure 13: Proposed scheme of HIV infected CD4+ T cells in absence **(a)** or presence **(b)** of the Nef device which induces XCL-1 production. Panel **(a)** illustrates that in the absence of the sensor, Nef induces HLA down-modulation and relocation to intracellular compartments, ensuing viral escape from the immune system. Panel **(b)** shows Nef sequestration by the device and subsequent effective HIV-peptides presentation by HLA to the immune system (CD8+ T cells and Dendritic cells DC). XCL-1 production induced by the device results in trafficking of the immune cells to the site of infection and its antiviral effect occur by binding the outer viral coat with subsequent block of HIV entry to the surrounding cells[24].

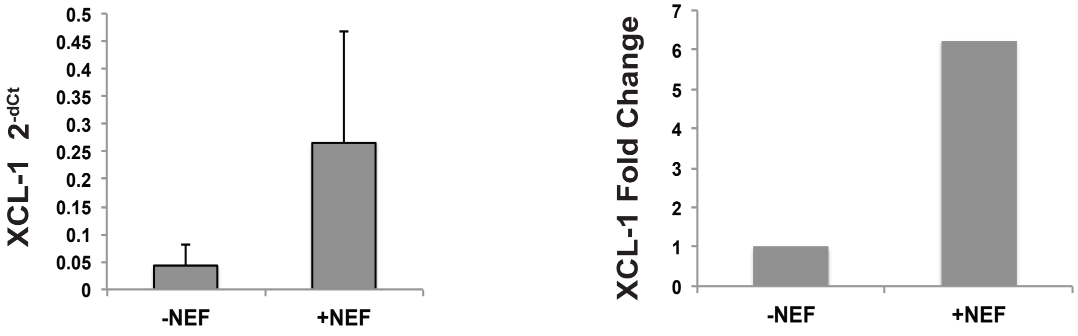

Supplementary Figure 14: XCL-1 expression is induced upon sensing Nef protein. Nef sensor-actuator device (LD0-TCS(L)/TEVp-LD0-SH3) was rewired to trigger XCL-1 chemokine expression. We transfected the device in 293FT HEK cells, in presence or absence of Nef. XCL-1 mRNA levels were measured by qPCR 48h post-transfection. qRT-PCR results were normalized to endogenous GAPDH mRNA levels, and are shown in the right panel as fold increase relative to negative control -Nef (n=3 independent technical replicates).

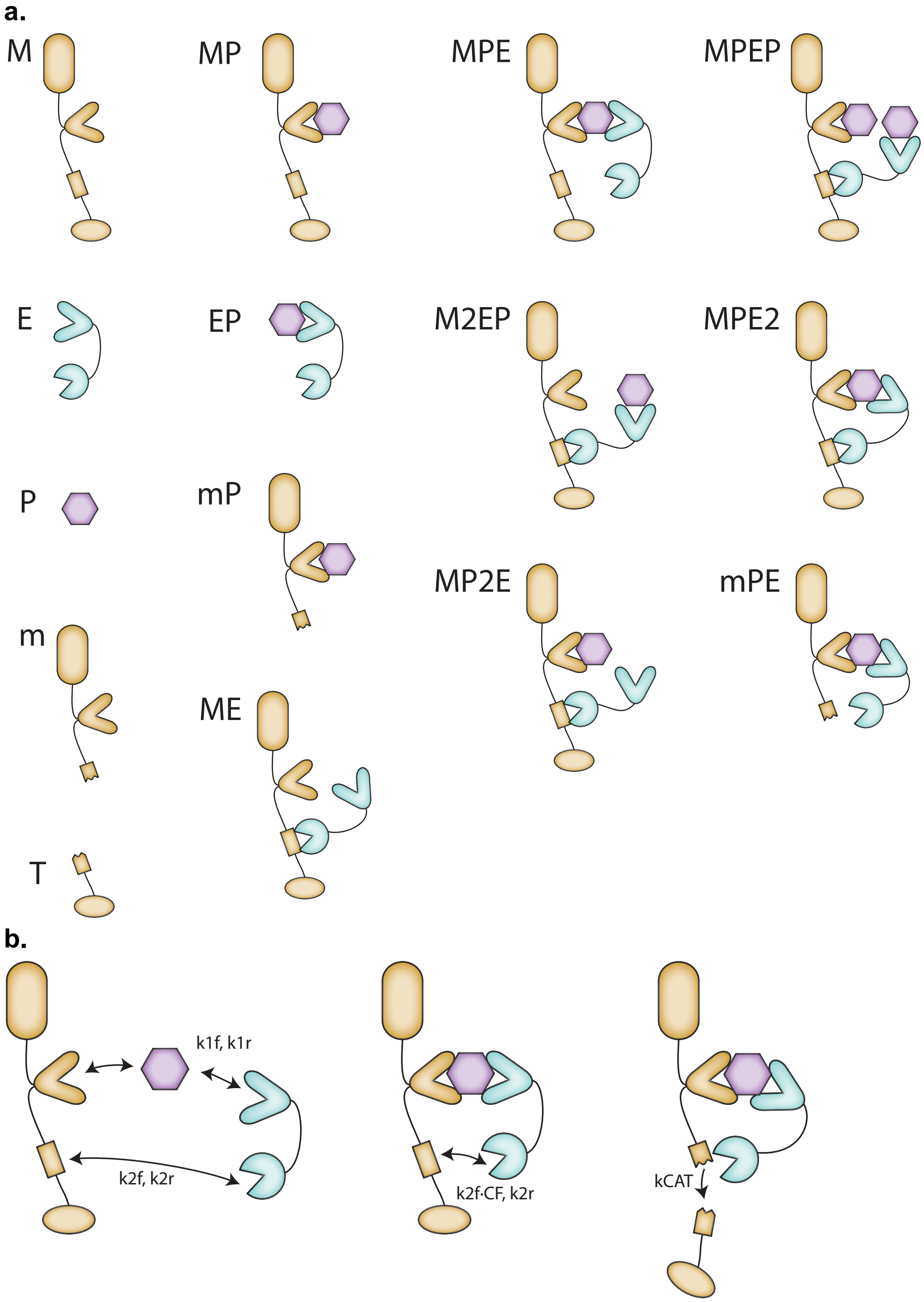

Supplementary Figure 15: Schematic diagrams of computational model. (a) The computational model consists of 15 different molecular species. (b) The sensor parts bind the protein of interest reversibly. TEV protease binds the TEV cleavage site (TCS) reversibly followed by irreversible cleavage of the TCS, which releases the transcription factor.

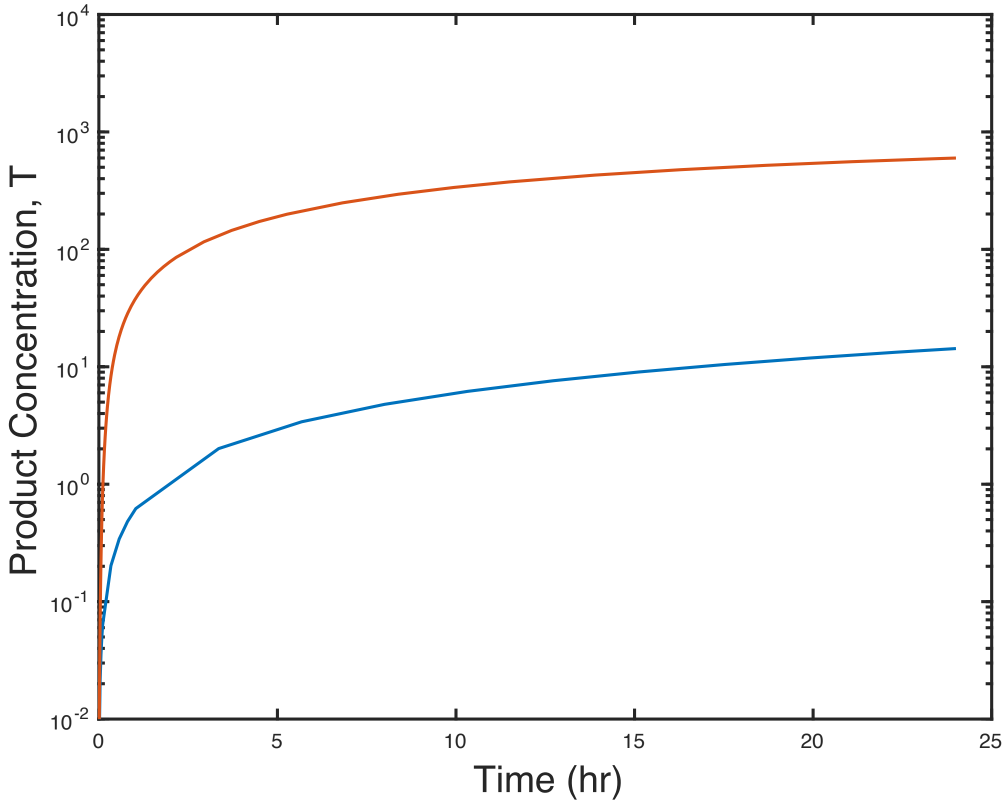

Supplementary Figure 16: Sample simulation trace for protein of interest = 0 pM (blue) and protein of interest = 1000 pM (red). Fold change is calculated at t = 24 hours for subsequent figures.

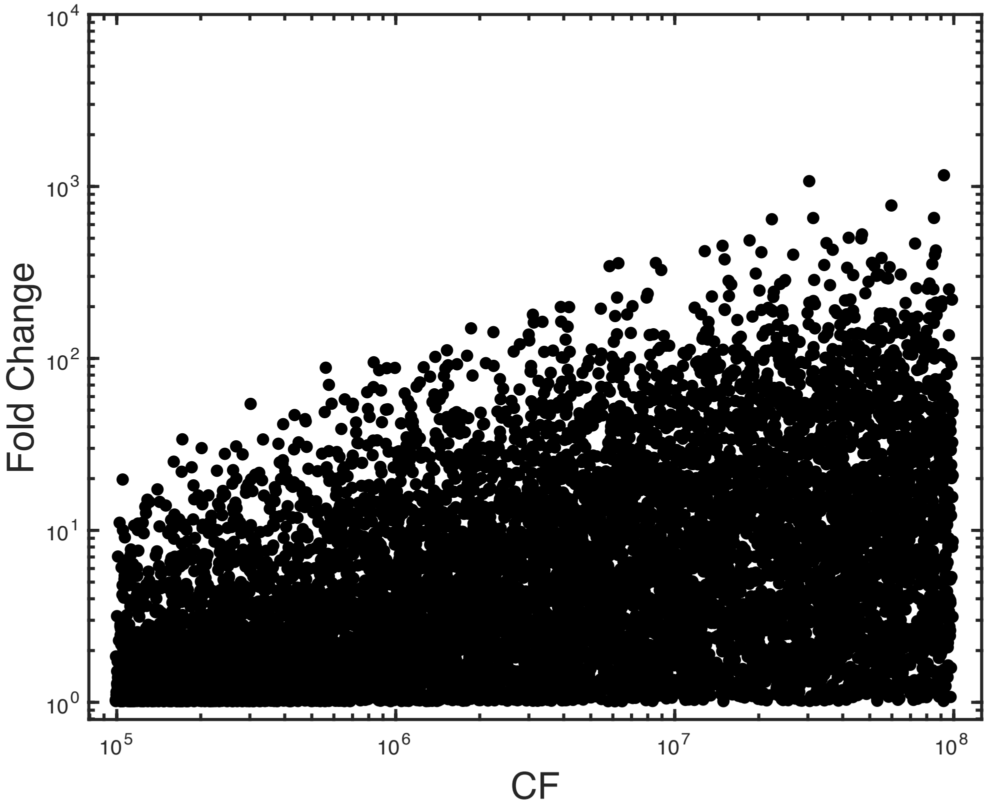

Supplementary Figure 17: Fold change plots for 10,000 simulations with randomly sampled parameter sets from the log-transformed realistic parameter spaces.

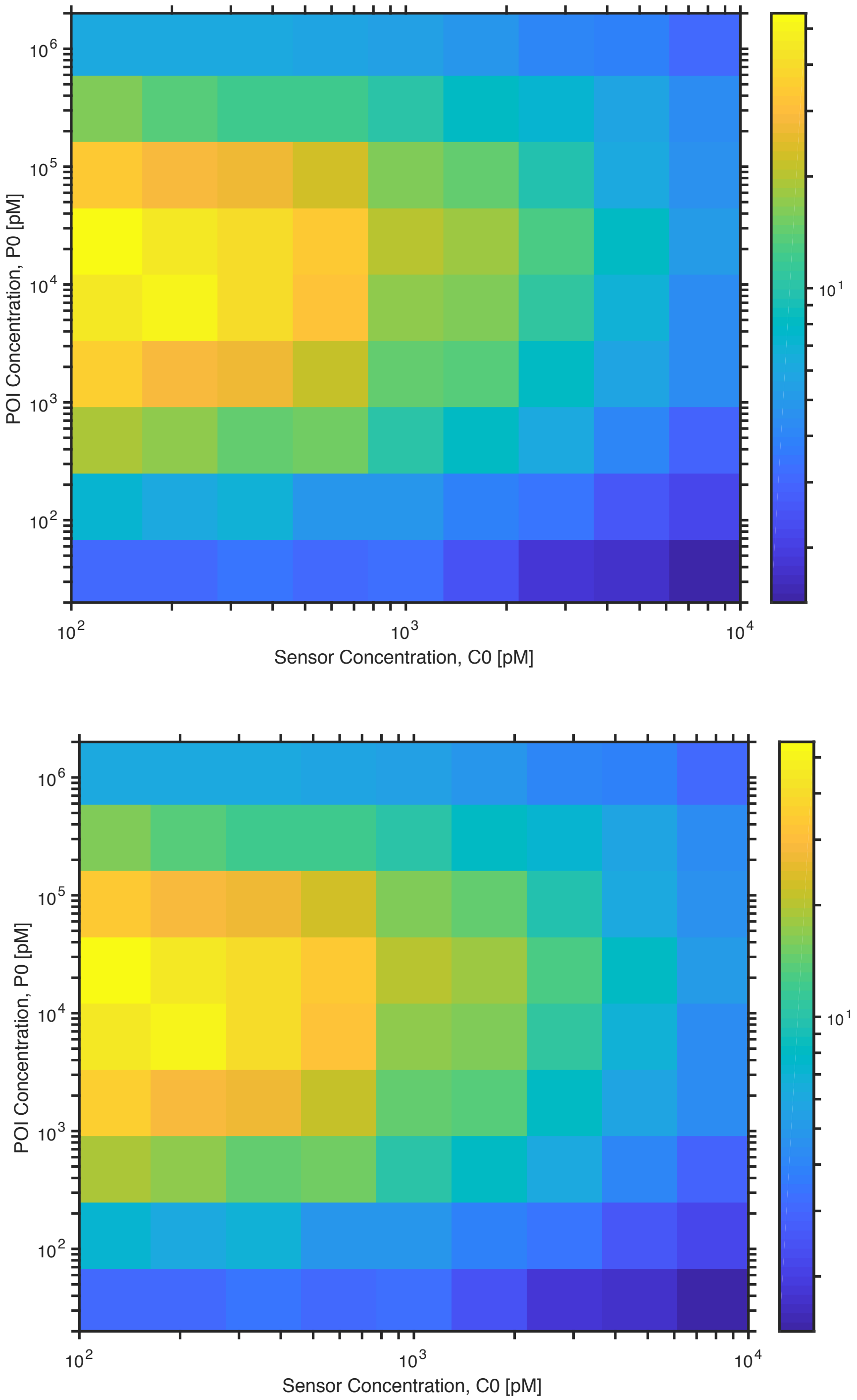

Supplementary Figure 18: Heat maps for the identification of parameter interactions, with color intensity indicating the geometric mean of fold change for each bin. CF was fixed at $2e^7$, while all other parameters were randomly sampled for 10,000 simulations.

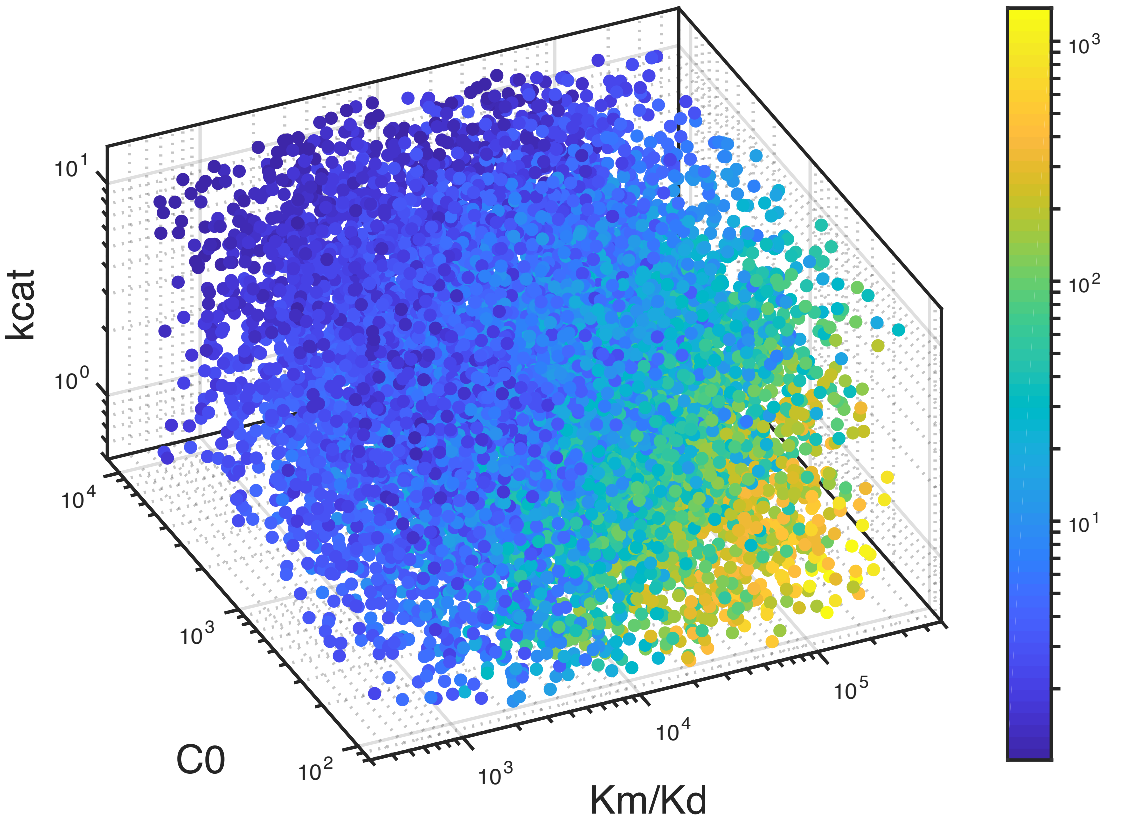

Supplementary Figure 19: To elucidate interactions between the remaining engineerable parameters, CF was fixed at $2e^7$ and P0 was fixed at $1e^3$ pM, while all other parameters were randomly sampled for 10,000 simulations. Color represents fold change.

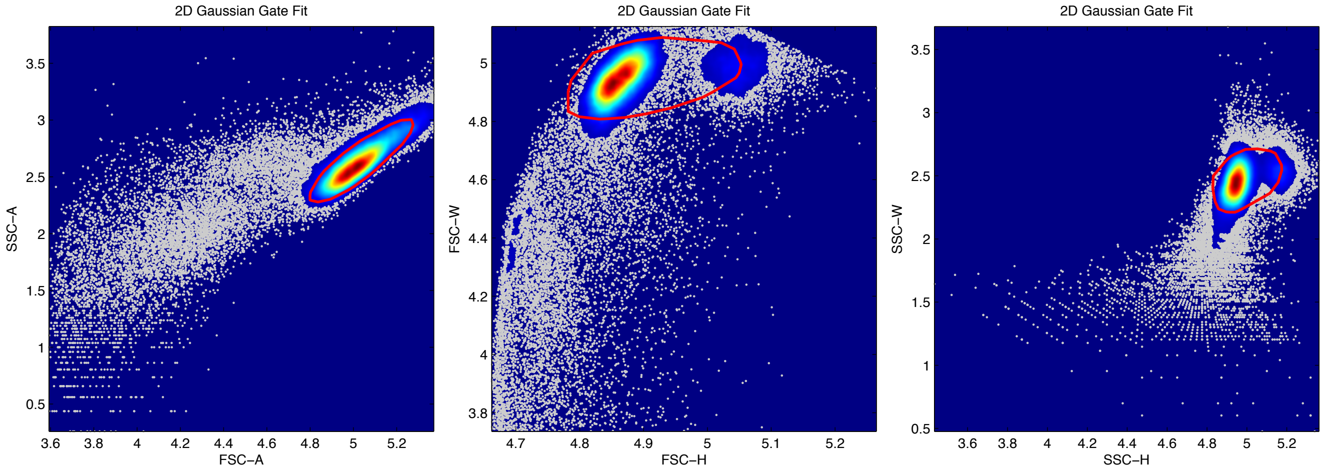

Supplementary Figure 20: example of population of live cells selected with flow cytometry according to FCS/SSC parameters.

Figure	Short plasmid name	Full plasmid name	Genbank accession codes
1&S4-6	pEXPR-16	16-pEXPR-Hef1a-nNS3	MH107778
S3-4	pEXPR-5	5-pEXPR-hEF1a-FGFR-mKate-ScFv35-LD15-TCS(L)-Gal4-Vp16	MH107777
S4	pEXPR-21	21-pEXRE-TRE_tight-DD-scFv162-LD15-Tev	MH107779
1&S4-6	pEXPR-37	37-pEXPR-TRE_tight-TEV-LD0-scFv162	MH107780
S4	pEXPR-38	38-pEXPR-TRE_tight-TEV-LD15-scFv162	MH107781
1&S3-6	pEXPR-39	39-pEXPR-Hef1a-FGFR-mKate-ScFv35-LD0-TCS(L)-Gal4-Vp16	MH107782
1&S5	pEXPR-1	1- pEXPR-TagBFP-LD15-NS3	MH225468
1-2&S3-5,7,9-11	pEXPR-UAS_G4	pEXPR-UAS_EYFP	MH225467
1&S6,8	pEXPR-43	43-pEXPR-UAS-hBAX	MH225455
1&S7,8	pEXPR-40	40-pEXPR-Hef1a-HTT(HDx1)	MH225456
1&S7,8	pEXPR-63	63-pEXPR-Hef1a-FGFR-mKate-Happ1-LD15-TCS(S)-Gal4-Vp16	MH107785
S7	pEXPR-64	64-pEXPR-hEF1A-FGFR-Mkate-Happ1-LD0-TCS(S)-G4-Vp16	MH225457
S7	pEXPR-65	65-pEXPR-TRE-TEV-LD15-VI12.1	MH107786
S7	pEXPR-66	66-pEXPR-TRE-TEV-LD0-VI12.1	MH225458
S7	pEXPR-71	71-pEXPR-TRE-DD-TEV-LD15-VI12.1	MH225459
1&S6,8	pEXPR-72	72-pEXPR-TRE-DD-TEV-LD0-VI12.1	MH225460
S9	pEXPR-49	49-pEXPR-Hef1a Tat	MH107784
S9	pEXPR-61	61-pEXPR-TRE_tight-scFv2-LD15-TEV	MH225461
S9	pEXPR-62	62-pEXPR-TRE_tight-scFv2-LD0-TEV	MH107783
S9	pEXPR-50	50-pEXPR-Hef1a-FGFR-mKate-scFv3-LD15-TCS(S)-G4-Vp16	MH107787
S9	pEXPR-51	51-pEXPR-Hef1a-FGFR-mKate-scFv3-LD15-TCS(L)-G4-Vp16	MH225462

S9	pEXPR-53	53-pEXPR-Hef1a-FGFR-mKate-scFv3-LD10-TCS(L)-G4-Vp16	MH225463
2&S10-12,14	pEXPR-156	156-pEXPR1_2-hEF1a-FGFR-mKate-sdAb19-LD0-TCS(L)-Gal4-Vp16	MH225464
2&S10-12,14	pEXPR-169	169-pEXPR2_3-TRE_tight-TEV2-LD0-SH3	MH225465
2	pEXPR-166	166-pEXPR1_2-hEF1a-FGFR-mKate-sdAb19-LD15-TCS(L)-Gal4	MH225466
2&S10	24162	pCI NL4-3 Nef-HA-WT(Addgene)	

Supplementary Table 1: List of plasmids used in this paper

	**Kd (nM)**
**scFv162**[32]	180
**scFv35**[32]	160
**Vl12.3**[33]	3nM
**Happ1**	N/A
**scFv2**	N/A
**scFv3**	N/A
**SH3**[34]	380
**sdAb19**[35]	2
	**Km (mM)**
**TCS-S**	0.043
**TCS-L**	0.240

Supplementary Table 2: Affinity of intrabodies used in our study and of TEVp towards the cleavage site. Of note, the efficiency of TEVp in processing a substrate containing the TEV Cleavage Sites (TCS) is affected by the amino acidic residue in P1', for which Km was previously determined[5].

Parameter	Description	Value		Source
K_D	Antibody Kd	1e3 : 5e5	pM	(approx. values in Suppl Table 2)
K_M	TEV K_M	4e7 : 4e8	pM	approx. values in Kapust et al[5]
k_{CAT}	TEV k_{CAT}	0.5 : 15	min^{-1}	approx. values in Kapust et al[5]
k_{1f}, k_{2f}	Association rates	1e-6 : 1e-4	$pM^{-1}min^{-1}$	Northrup et al[36]
$C0$	Sensor concentration	1e2 : 1e4	pM	
$P0$	POI concentration	1e2 : 2e6	pM	Colby et al[33]
CF	Enhanced binding factor	1e5 : 1e8		
k_{1r}	Antibody dissociation rate			$=K_D \cdot k_{1f}$
k_{2r}	TEV dissociation rate			$=K_M \cdot k_{1f} - k_{cat}$

Supplementary Table 3: Computational Model reaction rates

Supplementary References

1. Kapust, R. B. & Waugh, D. S. Controlled intracellular processing of fusion proteins by TEV protease. *Protein Expr. Purif.* **19,** 312–8 (2000).
2. Barnea, G. *et al.* The genetic design of signaling cascades to record receptor activation. *Proc. Natl. Acad. Sci. U. S. A.* **105,** 64–9 (2008).
3. Schwarz, K. A., Daringer, N. M., Dolberg, T. B. & Leonard, J. N. Rewiring human cellular input–output using modular extracellular sensors. *Nat. Chem. Biol.* **13,** 202–209 (2016).
4. Wieland, M. *et al.* Engineered UV-A light-responsive gene expression system for measuring sun cream efficacy in mammalian cell culture. *J. Biotechnol.* **189,** 150–153 (2014).
5. Kapust, R. B., Tözsér, J., Copeland, T. D. & Waugh, D. S. The P1' specificity of tobacco etch virus protease. *Biochem. Biophys. Res. Commun.* **294,** 949–55 (2002).
6. Hammers, C. M. & Stanley, J. R. Antibody phage display: technique and applications. *J. Invest. Dermatol.* **134,** e17 (2014).
7. Boder, E. T. & Wittrup, K. D. Yeast surface display for screening combinatorial polypeptide libraries. *Nat. Biotechnol.* **15,** 553–7 (1997).
8. Patel, C. A. *et al.* Parallel selection of antibody libraries on phage and yeast surfaces via a cross-species display. *Protein Eng. Des. Sel.* **24,** 711–9 (2011).
9. Harmsen, M. M. & De Haard, H. J. Properties, production, and applications of camelid single-domain antibody fragments. *Appl. Microbiol. Biotechnol.* **77,** 13–22 (2007).
10. Robinson, C. R. & Sauer, R. T. Optimizing the stability of single-chain proteins by linker length and composition mutagenesis. *Proc. Natl. Acad. Sci.* **95,** 5929–5934 (1998).
11. Southwell, A. L. *et al.* Intrabodies binding the proline-rich domains of mutant huntingtin increase its turnover and reduce neurotoxicity. *J. Neurosci.* **28,** 9013–9020 (2008).
12. Romani, B., Engelbrecht, S. & Glashoff, R. H. Functions of Tat: the versatile protein of human immunodeficiency virus type 1. *J. Gen. Virol.* **91,** 1–12 (2010).
13. Le Douce, V. *et al.* Achieving a cure for HIV infection: do we have reasons to be optimistic? *J. Antimicrob. Chemother.* **67,** 1063–74 (2012).
14. Siliciano, J. M. & Siliciano, R. F. The Remarkable Stability of the Latent Reservoir for HIV-1 in Resting Memory CD4 + T Cells. doi:10.1093/infdis/jiv219
15. Mann, J. K. *et al.* Ability of HIV-1 Nef to downregulate CD4 and HLA class I differs among viral subtypes. *Retrovirology* **10,** 100 (2013).
16. Pereira, E. A. & daSilva, L. L. P. HIV-1 Nef: Taking Control of Protein Trafficking. *Traffic* **17,** 976–996 (2016).
17. Sloan, R. D., Donahue, D. A., Kuhl, B. D., Bar-Magen, T. & Wainberg, M. A. Expression of Nef from unintegrated HIV-1 DNA downregulates cell surface CXCR4 and CCR5 on T-lymphocytes. *Retrovirology* **7,** 44 (2010).
18. Wu, Y. & Marsh, J. W. Selective transcription and modulation of resting T cell activity by preintegrated HIV DNA. *Science* **293,** 1503–6 (2001).
19. Hartung, E. *et al.* Induction of potent CD8 T cell cytotoxicity by specific targeting of antigen to cross-presenting dendritic cells in vivo via murine or human XCR1. *J. Immunol.* **194,** 1069–79 (2015).
20. Cairns, C. M. *et al.* Lymphotactin expression by engineered myeloma cells drives tumor regression: mediation by CD4+ and CD8+ T cells and neutrophils

expressing XCR1 receptor. *J. Immunol.* **167,** 57–65 (2001).
21. Terhorst, D. *et al.* Laser-Assisted Intradermal Delivery of Adjuvant-Free Vaccines Targeting XCR1+ Dendritic Cells Induces Potent Antitumoral Responses. *J. Immunol.* **194,** 5895–5902 (2015).
22. Holliger, P. & Hudson, P. J. Engineered antibody fragments and the rise of single domains. *Nat. Biotechnol.* **23,** 1126–36 (2005).
23. Dorner, B. G. *et al.* Selective Expression of the Chemokine Receptor XCR1 on Cross-presenting Dendritic Cells Determines Cooperation with CD8+ T Cells. *Immunity* **31,** 823–833 (2009).
24. Guzzo, C. *et al.* The CD8-Derived Chemokine XCL1/Lymphotactin Is a Conformation-Dependent, Broad-Spectrum Inhibitor of HIV-1. *PLoS Pathog.* **9,** 1–11 (2013).
25. Fox, J. C. *et al.* Engineering Metamorphic Chemokine Lymphotactin/XCL1 into the GAG-Binding, HIV-Inhibitory Dimer Conformation. *ACS Chem. Biol.* **10,** 2580–2588 (2015).
26. Jacobs, E. S. *et al.* Cytokines Elevated in HIV Elite Controllers Reduce HIV Replication In Vitro and Modulate HIV Restriction Factor Expression. *J. Virol.* **91,** JVI.02051-16 (2017).
27. Shacklett, B. L. & Anton, P. a. HIV Infection and Gut Mucosal Immune Function: Updates on Pathogenesis with Implications for Management and Intervention. *Curr. Infect. Dis. Rep.* **12,** 19–27 (2010).
28. MATLAB and Statistics Toolbox Release 2012b, The MathWorks, Inc., Natick, Massachusetts, United States.
29. Jencks, W. P. On the attribution and additivity of binding energies. *Biochemistry* **78,** 4046–4050 (1981).
30. Lauffenburger, D. A. & Linderman, J. J. *Receptors : models for binding, trafficking, and signaling*. (Oxford University Press, 1993).
31. Beal, J. Signal-to-Noise Ratio Measures Efficacy of Biological Computing Devices and Circuits. *Front. Bioeng. Biotechnol.* **3,** 93 (2015).
32. Gal-Tanamy, M. *et al.* HCV NS3 serine protease-neutralizing single-chain antibodies isolated by a novel genetic screen. *J. Mol. Biol.* **347,** 991–1003 (2005).
33. Colby, D. W. *et al.* Potent inhibition of huntingtin aggregation and cytotoxicity by a disulfide bond-free single-domain intracellular antibody. *Proc. Natl. Acad. Sci. U. S. A.* **101,** 17616–21 (2004).
34. Lee ', C.-H. *et al.* A single amino acid in the SH3 domain of Hck determines its high affinity and specificity in binding to HIV-1 Nef protein. *EMBO J.* **14,** 5006–5015 (1995).
35. Bouchet, J. *et al.* Inhibition of the Nef regulatory protein of HIV-1 by a single-domain antibody. *Blood* **117,** 3559–3568 (2011).
36. Northrup, S. H. & Erickson, H. P. Kinetics of protein-protein association explained by Brownian dynamics computer simulation. *Proc. Natl. Acad. Sci. U. S. A.* **89,** 3338–42 (1992).
